# Proinflammatory and GABA eating bacteria in Parkinson's disease gut microbiome from a meta-analysis perspective

**DOI:** 10.1038/s41531-025-00950-z

**Published:** 2025-06-03

**Authors:** Nour H. Marzouk, Hannah H. Rashwan, Mohamed El-Hadidi, Raghda Ramadan, Mohamed Mysara

**Affiliations:** 1 Bioinformatics Group, Centre for Informatics Science (CIS), School of Information Technology and Computer Science (ITCS), Nile University, Giza, Egypt; 2 School of Biotechnology, Nile University, Giza, Egypt; 3 Present Address: Cancer and Genomic Sciences, School of Medical Sciences, College of Medicine and Health (CME), University of Birmingham Dubai, Dubai, United Arab Emirates

**Keywords:** Parkinson's disease, Diagnostic markers, Predictive markers, Predictive medicine, Microbiology

## Abstract

Parkinson’s disease (PD) is the second most common neurodegenerative disorder, characterized by motor dysfunction coupled with gastrointestinal disturbances. Recent studies implicate the gut microbiome with the development of PD, yet pinpointing the exact microbial players is still to be determined. This meta-analysis is the first to consolidate five homogenous case-control studies, covering the same variable regions of the 16S rRNA of 1007 fecal samples. Utilizing our unified pipeline, we identified several key players potentially contributing to PD. Our findings reveal higher microbial diversity characterized by elevated levels GABA consuming species particularly *Evtepia gabavorous*, contributing to neuronal excitability. We also report the abundance of the proinflammatory *Klebsiella variicola* and the H_2_S-producing *Streptococcus anginosus* bacteria, potentially promoting α-synuclein accumulation in the brain. This comprehensive analysis highlights the potential of gut microbiota as a biomarker and a therapeutic strategy to mitigate the progression of PD, possibly facilitating diagnosis and enhancing patient outcomes.

## Introduction

Parkinson’s disease (PD) is the second most common age-related neurodegenerative disorder (NDD), affecting over 10 million people worldwide as of 2020, which is expected to increase by 56% by 2030^1,2^. It is primarily characterized by motor dysfunction, often initially manifesting as tremor, balance issues, rigidity, and bradykinesia^3^. As the disease progresses, symptoms expand to gastrointestinal disturbances such as gastroparesis, gut inflammation, increased intestinal permeability, and constipation^4^. These symptoms are largely attributed to the accumulation of Lewy bodies in addition to misfolded α-synuclein (αSyn) protein aggregates, which is one of the PD hallmarks and a leading cause of neuronal toxicity and death^5^. This is accompanied by the degeneration of dopaminergic neurons and a subsequent reduction in dopamine levels, which are the primary molecular mechanisms responsible for the motor impairments in PD^6^. Additionally, mitochondrial dysfunction, oxidative stress, neuroinflammation, and impaired protein degradation pathways further contribute to disease progression^7^.

Given the early involvement of the gastrointestinal tract, which often precedes motor symptoms by several years, alterations in gut microbiota composition have been widely investigated in the context of PD pathophysiology^8^. Studies support the potential role of gut microbiota in PD, demonstrating that the microbiota can influence αSyn deposition, which may begin in the neurons of the intestinal submucosa up to 8 years before motor symptoms emerge^9,10^. Altered gut microbiota composition may trigger immune activation through a compromised gut barrier, leading to systemic inflammation, which can disrupt the blood-brain barrier (BBB), promote neuroinflammation, and contribute to neuronal degeneration^11^. Intestinal inflammation triggered by bacterial pathobionts may also initiate αSyn misfolding, which can reach the brain through the vagus nerve^12^.

Another pivotal player in PD is the *Gamma*-aminobutyric acid (GABA), the brain’s primary inhibitory neurotransmitter, which plays a critical role in the disease pathophysiology^13–15^. Increasing evidence highlights a dynamic interplay between gut-derived GABA and its circulating and cerebral levels, underscoring the microbiome’s potential influence on neurological health^16–18^. Certain gut bacterial species can consume or produce GABA, thereby modulating its systemic availability. The work of Strandwitz et al. has identified *Evtepia gabavorous* as a GABA-consuming species that thrives in the presence of *Bacteroides fragilis*, a potent GABA producer^16^. Their study also revealed numerous candidate GABA-producing and -consuming species, underscoring the complex microbial networks involved in GABA metabolism. Another interesting work by Wu et al. has demonstrated that supplementation with GABA-producing bacterial strains can significantly alleviate essential tremors via the gut-brain axis, particularly through the enteric nervous system and vagus nerve pathways^19^. These findings suggest the microbiota-derived GABA may influence brain function and potentially contribute to PD progression. The growing interest in the gut–brain axis has led to extensive research into gastrointestinal pathology and gut microbiota alterations in patients with PD. Numerous studies have linked the disease with an increased abundance of specific gut microbiota, such as the Akkermansia genus and the Verrucomicrobiaceae family^12^. However, some findings remain contested, such as the reported enrichment of Lactobacillaceae in Western cohorts but not in Chinese populations, along with inconsistent results regarding the role of Prevotellaceae^20–22^. Moreover, there remains limited consensus on the specific bacterial species and their underlying mechanisms that may contribute to the PD pathophysiology^23^. Discrepancies among studies may arise from variations in study designs and methods used for the data analysis, as well as the inherent variability of gut microbiota across populations, lifestyles, and diets. These factors make it challenging to identify specific intestinal bacteria contributors to the pathogenesis of PD.

This variability has underscored the necessity for a comprehensive meta-analysis to synthesize and reconcile the diverse findings across studies^4,24–29^. Several meta-analyses have explored the gut microbiome in PD, and variations in alpha and beta diversity across studies have been noted. However, these analyses have not adequately resolved the inconsistencies or identified the specific microbial species that may contribute to the disease^23,30–36^. Examples of heterogeneity in previous meta-analyses include variations in sample collection, variable sections of 16S rRNA regions, and sequencing strategies used in these meta-analyses^23,30–32,35^. Some meta-analyses only presented summary statistics without analyzing raw data, potentially overlooking methodological biases^36^. Selecting the V3 region over V4 has been questioned due to heterogeneity, even though combining regions V3-V4 tends to yield more accurate results^30,32,37^. Furthermore, integrating shotgun and amplicon sequencing in meta-analyses has been problematic, as few studies have successfully merged the advantages of both technologies due to a lack of dataset comparability and standardized protocols^30^. Challenges in accessing raw data have led researchers to resort to tools like GetData Graph Digitizer, which may introduce bias^35^.

Our meta-analysis aims to address study heterogeneity by standardizing methodology and minimizing potential biases arising from varied data processing techniques. This was achieved by applying a standardized workflow across all samples, incorporating a compositionality-aware method to correct for artefactual variations in the relative abundances of bacterial species within the microbiome. By carefully selecting studies with the highest degree of homogeneity, we focused on paired-end 16S rRNA sequencing of the V3-V4 regions of the gut microbiota from PD patients without prior antibiotic administration. This stringent selection strategy ensured consistency across the dataset and minimized variability while confirming the PD diagnosis. As a result, our meta-analysis is uniquely comprehensive, offering a robust foundation for early PD detection through gut microbiome profiling.

## Results

### Systematic search results and quality control on the selected studies

**Fig. 1:**
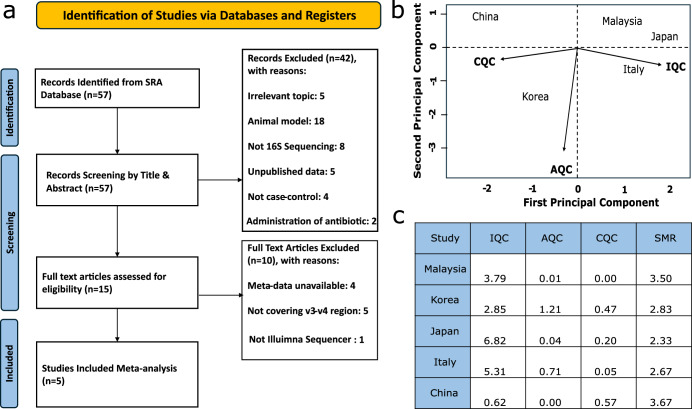
Systematic search selected studies and their quality control results. **a** PRISMA chart illustrating studies removed for being irrelevant or heterogeneous for the meta-analysis. **b** PCA biplot of QC measures (Internal Quality Control (IQC), Accuracy Quality Control (AQC), Consistency Quality Control (CQC), and Standardized Mean Rank (SMR)) demonstrating that the 5 studies are conceding with at least one of these quality measures of MetaQC. **c** Summary table of MetaQC results covering IQC, AQC, CQC, and SMR where the high score represents high consistency and the low one represents low consistency.

Our comprehensive initial search yielded 57 records from the SRA database, which were then carefully screened for relevance and quality (Supplementary Table 1). We identified 15 primary case-control studies addressing the PD-associated gut microbiome using 16S rRNA gene amplicon sequencing data. Upon further review, 10 articles were excluded due to various inconsistencies, ultimately leaving us with the five most homogenous studies, which were included in our meta-analysis (Fig. 1a). These studies covered cohorts from Japan, Malaysia, Italy, South Korea, and China.

In order to assess the level of the heterogeneity among the included studies and to assess the presence of any outlier studies, we performed rigorous quality control using MetaQC^38^. From the results, all studies were consistent with at least one of the consistency measurements, namely IQC, AQC, and CQC. This was also reflected in the overall Standardized Mean Rank (SMR), which measures overall consistency, indicating that none of the cohorts showed inconsistency (Fig. 1b, c).

Collectively, the five cohorts provided a total of 1007 samples after filtration, all derived from case–control studies (Table 1). Sequencing technologies used across these studies included MiSeq, HiSeq, and NovaSeq platforms, ensuring comprehensive microbial profiling. Cases had an average age ranging between 55.65 and 78.1 years across all studies, while controls’ ages ranged from 55.4 to 78.1 years. To assess the microbial difference between the cohorts, we used the control samples within each study. The results showed a significant difference between the cohorts’ alpha diversity in both inverse simpson and observed richness indices (*P* < 0.05; Kruskal-Wallis) among their baselines. Also, beta-diversity analysis revealed a significant difference between the cohorts (*P* < 0.05; Kruskal-Wallis). However, when observing the over-represented species as well as under-represented species taking the China sample as baseline, we reported 24 differentially abundant OTUs, as shown in (Supplementary Fig. 1). This approach was also applied to the PD samples to measure the differences across the studies, where a significant difference was observed between the cohorts’ alpha diversity (*P* < 0.05; Kruskal-Wallis), and the beta-diversity analysis was also significant, but only 11 significant OTUs were identified among the PD samples (Supplementary Fig. 2).

**Table 1:** Studies included in the meta-analysis

Country	Numbers	Seq. Tech.	Accession number	Study
Japan	209 PD & 104 HC	16S rRNA MiSeq	PRJDB8639	^25^
Malaysia	103 PD & 96 HC	16S rRNA HiSeq	PRJNA494620	^26^
Italy	118 PD & 84 HC	16S rRNA Miseq	PRJNA510730	^24^
South Korea	88 PD & 84 HC	16S rRNA Miseq	PRJNA742875	^4^
China	67 PD & 54 HC	16S rRNA Novaseq	PRJNA822998	^27^

The table presents an overview of research studies examining the gut microbiome of PD. The country where each study was conducted, the number of HC and PD participants involved, the sequencing technology (Seq. Tech.) and type used, the accession number for the data included.

### Variation in microbial composition and increased diversity in Parkinson’s disease versus controls

**Fig. 2:**
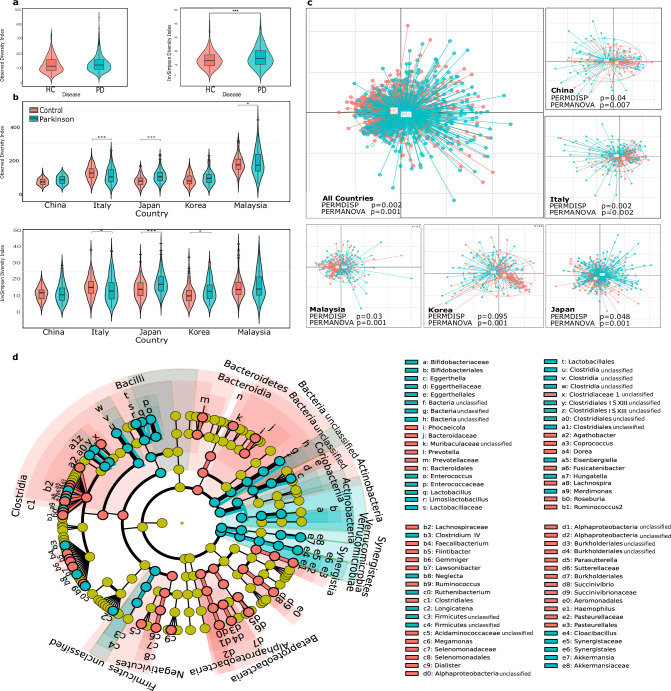
Comparative analysis of microbial diversity and composition in Parkinson’s disease (PD) and Healthy Control (HC). **a** Observed Richness and Inverse Simpson Index, assessing species diversity across different classes. **b** Observed Richness and Inverse Simpson Index, depicting the estimated variety of microbial species across groups and within individual studies. **c** Beta diversity analysis, comparing the overall composition of microbial communities between PD and HC samples, both across all studies and within each individual study. **d** A cladogram showing significant bacteria across various taxonomic levels, offering a hierarchical perspective of microbial differences.

Our in-depth analysis of gut microbiomes from PD patients and Healthy Control (HC) included comprehensive assessments of alpha and beta diversities along with microbial abundance across the five included studies. The alpha diversity, measured by the inverse simpson index, showed a significant increase (*P* < 0.05) in microbial diversity within the PD gut microbiome compared to HC. On the other hand, the observed richness index did not show significant differences between the two groups (Fig. 2a). When examined across individual studies, significant differences for both the inverse simpson and observed richness indices were also observed in the studies from Italy and Japan (Fig. 2b).

The beta diversity analysis was first conducted using the Rhea pipeline, combining data from all studies using unweighted UniFrac distances. Although the combined analysis of the five studies indicated significant results (*P* < 0.05; PERMANOVA; Fig. 2c), no clear separation between both clusters appeared. This might be attributed to the fact that the inter-study variability overshadowed the differences between PD and HC. To address this, each study was analyzed separately, revealing clear distinctions between the two conditions. This was further confirmed using RCM to account for the influence of study-specific effects (Supplementary Fig. 3a, b) and when accounting for the study effect (Supplementary Fig. 3c, d).

In our analysis, microbial abundance was quantified, identifying different taxonomic levels with significant differences between PD samples and HC, as determined by ANCOM-BC with a False Discovery Rate (FDR) below 0.05. The cladogram showed that the phyla Bacteroidetes and Synergistetes were significantly upregulated. In the phylum Firmicutes, the class Negativicutes was found to be downregulated, whereas Bacilli and Clostridia showed upregulation. The phylum Verrucomicrobia, including its class Verrucomicrobiae, as well as the phylum Actinobacteria and its class Coriobacteriia, experienced upregulation. Furthermore, the classes Betaproteobacteria and Alphaproteobacteria within the phylum Proteobacteria and the class Synergistia within the phylum Synergistetes were also upregulated (Fig. 2d, detailed table available in Supplementary Table 2).

### Identification of bacterial profiles as potential biomarkers in the Parkinson’s disease microbiome

Through our in-depth analysis, species that were differentially abundant between PD patients and HC were confirmed by both ANCOM-BC and MetaDE analyses. ANCOM-BC is particularly adept at handling the compositional nature of microbiome data, accounting for potential biases that traditional differential abundance methods might overlook. Using ANCOM-BC, we were able to identify 138 species, highlighting its robust sensitivity in detecting differentially abundant taxa. MetaDE, on the other hand, aggregates data across multiple studies, enhancing the statistical power and reliability of the findings through combined effect sizes (detailed list available in Supplementary Table 3). Ten species showed significant upregulation in PD, including *Bifidobacterium longum, Bifidobacterium bifidum, Bifidobacterium pseudocatenulatum, Streptococcus anginosus, Lactobacillus acidophilus, Evtepia gabavorous, Mordavella massiliensis, Eubacterium siraeum, Klebsiella variicola*, and *Parabacteroides goldsteinii*. Additionally, nine species were significantly downregulated in PDs, including *Lachnoclostridium edouardi, Clostridium fessum, Dialister invisus, Coprococcus eutactus*, and *Lactobacillus rogosae* (FDR < 0.05; Fig. 4a, b & c).

Building upon these species-specific findings, further analysis revealed that these changes extended to higher taxonomic levels, impacting entire classes and phyla. The species *C.* *eutactus*, *L.* *edouardi*, and *C.* *fessum* within the Clostridia class of the Firmicutes phylum were significantly downregulated. Conversely, the species *A.* *butyriciproducens* from the Ruminococcaceae family in the same phylum was upregulated. Species within the genus Bifidobacterium of the Actinobacteria phylum were consistently upregulated across all levels, from species to phylum. Similarly, in the Bacteroidetes phylum, species such as *P.* *goldsteinii* within the Bacteroidia class and Bacteroidales order were notably upregulated. Furthermore, within the Proteobacteria phylum, the species *K.* *variicola* in the Gammaproteobacteria class and Enterobacteriaceae family were significantly upregulated. In contrast, the Firmicutes phylum showed a decrease, especially notable within the Clostridia class where numerous families, including Lachnospiraceae and Clostridiaceae, were markedly downregulated.

**Fig. 3:**
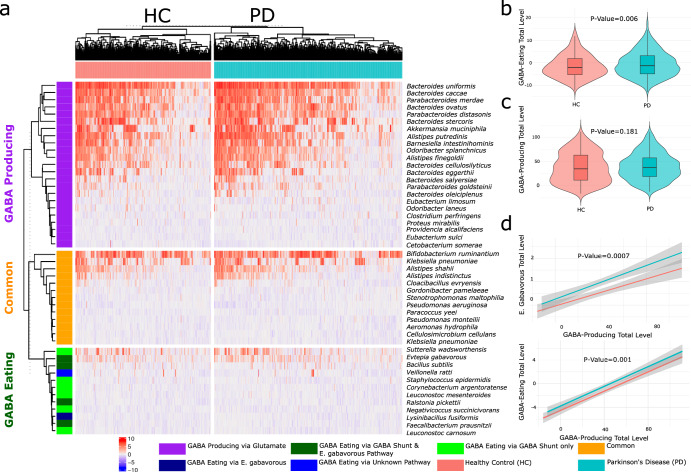
GABA-modulating bacteria analysis in Parkinson’s disease (PD) and healthy control (HC). **a** Heatmap illustrating the distribution of GABA-producing, GABA-eating, and common (both producing and eating) species. **b** Violin plots comparing the abundance of GABA-eating species between Parkinson’s disease (PD) and Healthy controls (HC). **c** Violin plots comparing the abundance of GABA-producing bacterial species between Parkinson’s disease (PD) and Healthy control (HC). **d** Linear model showing the relationship between the total GABA-eating species and the total GABA-producing species.

Among the detected species, we have identified several GABA producers, consumers, and common species capable of both consuming and producing GABA from the collection reported in the work of Strandwitz et al.^16^. When looking into the 23 identified GABA producers, we are not able to report any significant difference between PD and HC neither on the overall producer load (Fig. 3c, *P* > 0.18, Mann-Whitney) nor on the individual species abundance (with exception of the *P. goldsteinii* species; FDR = 0.049; meta-DE). Regarding the 18 GABA consumers detected, PD samples demonstrated a significant increase in the overall microbial load (Fig. 3b, *P* < 0.006, Mann-Whitney), particularly *E.* *gabavorus* a potent GABA eating species (FDR = 0.003; meta-DE). This was further confirmed when correlating over all GABA-consuming levels with other GABA-producing species within our cohorts. The results demonstrated an increase in the microbial load of GABA consumers with an increase in the GABA producers within HC samples (P < 0.05; *lm*; Fig. 3d). Yet, the GABA consumers are present at a higher abundance in the PD samples throughout the different levels of the GABA producers (*P* < 0.001; *lm*; Fig. 3d). This was also confirmed when modeling *E. gabavorus* levels to the overall GABA producers (*P* < 0.0007; *lm*; Fig. 3d) and with each one independently (Supplementary Figure 4).

### Gut microbiome functional pathways associated with Parkinson’s disease in patients and controls

**Fig. 4:**
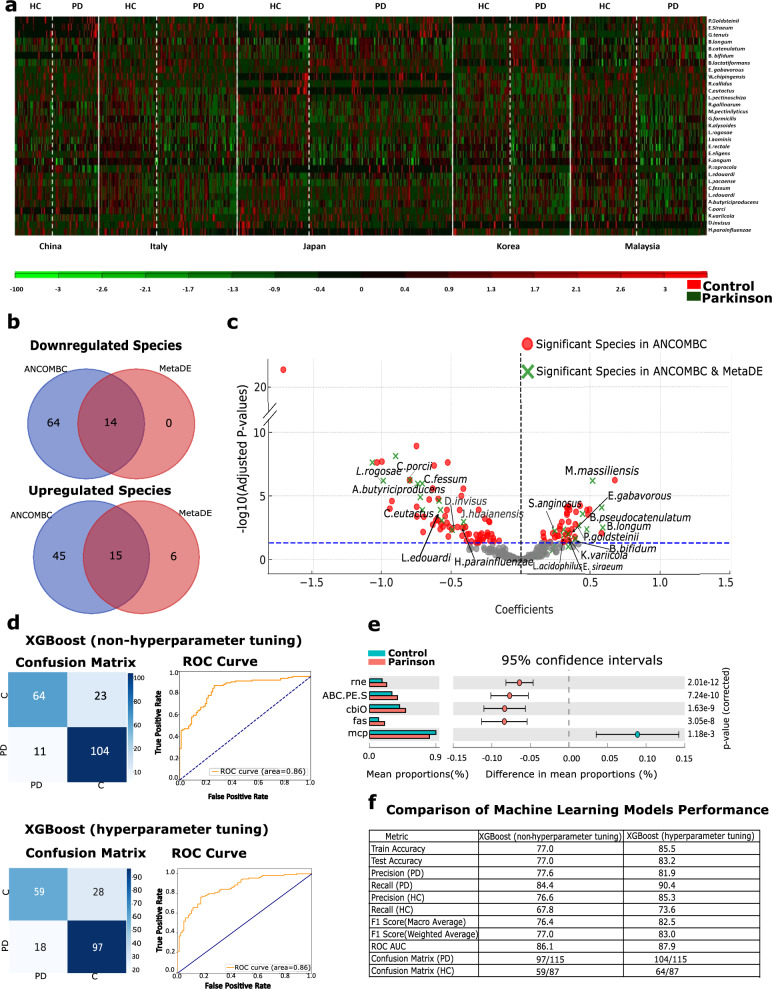
Comprehensive visualization of bacterial relevance and predictive modeling in Parkinson’s disease (PD) and Healthy Control (HC). **a** heatmap illustrating the significant bacterial species with differential abundance between Parkinson’s Disease (PD) and Healthy Control (HC), providing a clear visual representation of microbial alterations associated with PD. **b** Venn diagram showing the overlap of significant species identified as both upregulated and downregulated in PD patients by ANCOM-BC and MetaDE. **c** Volcano plot of significant species in PD as identified by ANCOM-BC, with green labels denoting species common between ANCOM-BC and MetaDE analyses. **d** Confusion matrices and Receiver Operating Characteristic (ROC) curves for machine learning models. **e** Results from STAMP, highlighting key functional pathways affected in PD, which may offer insights into potential mechanisms of disease progression. **f** Table of performance metrics, showcasing the predictive accuracy and effectiveness of these models in differentiating between PD and HC samples.

Our functional analysis, performed using STAMP, revealed five significantly enriched KOs in PD samples (Fig. 4e). Among these KOs, fatty acid synthase is involved in multiple metabolic pathways such as fatty acid biosynthesis, mycolic acid biosynthesis, fatty acid metabolism, and insulin resistance. The peptide/nickel transport system substrate-binding protein was linked to quorum sensing, while the cobalt/nickel transport system ATP-binding protein was associated with ABC transporter pathways. Lastly, ribonuclease E was identified as a key player in RNA degradation.

Further association analysis using MicrobiomeAnalyst revealed substantial differences between PD patients and HC in several metabolic pathways. Notably, several pathways such as Glycerolipid metabolism, Valine, Leucine, and Isoleucine degradation, Glycine, Serine, and Threonine metabolism, and Sulfur metabolism were significantly enriched in PD (FDR < 0.05, detailed list available in Supplementary Table 4).

### Effectiveness of machine learning models in differentiating Parkinson’s disease patients from healthy individuals using gut microbiota

We developed a machine learning model for classifying samples into two diagnostic categories: PD and HC. The feature selection was based on correlation with these categories, resulting in an optimized and regularized XGBoost model that demonstrated high accuracy and generalizability. A final set of 40 features at the genus level, 18 at the species level, 13 at the family level, 10 at the order level, 4 at the class level, and 1 at the phylum level. Additionally, some geographic and demographic factors were incorporated to ensure a comprehensive representation of the dataset. The accuracy scores of the XGBoost model achieved 77.0% for training accuracy and 77.2% for testing accuracy, indicating better generalizability in predicting PD. Furthermore, grid search cross-validation was conducted on the XGBoost model to optimize its hyperparameters. Using the best parameters, with the L1 regularization parameter set to 0.9 and the L2 regularization parameter set to 0.6, the model achieved a training accuracy of 86.5% and a testing accuracy of 83.2% (Fig. 4d). These results suggest that the optimized and regularized XGBoost model performed well on both the training and test datasets (Fig. 4f).

## Discussion

**Fig. 5:**
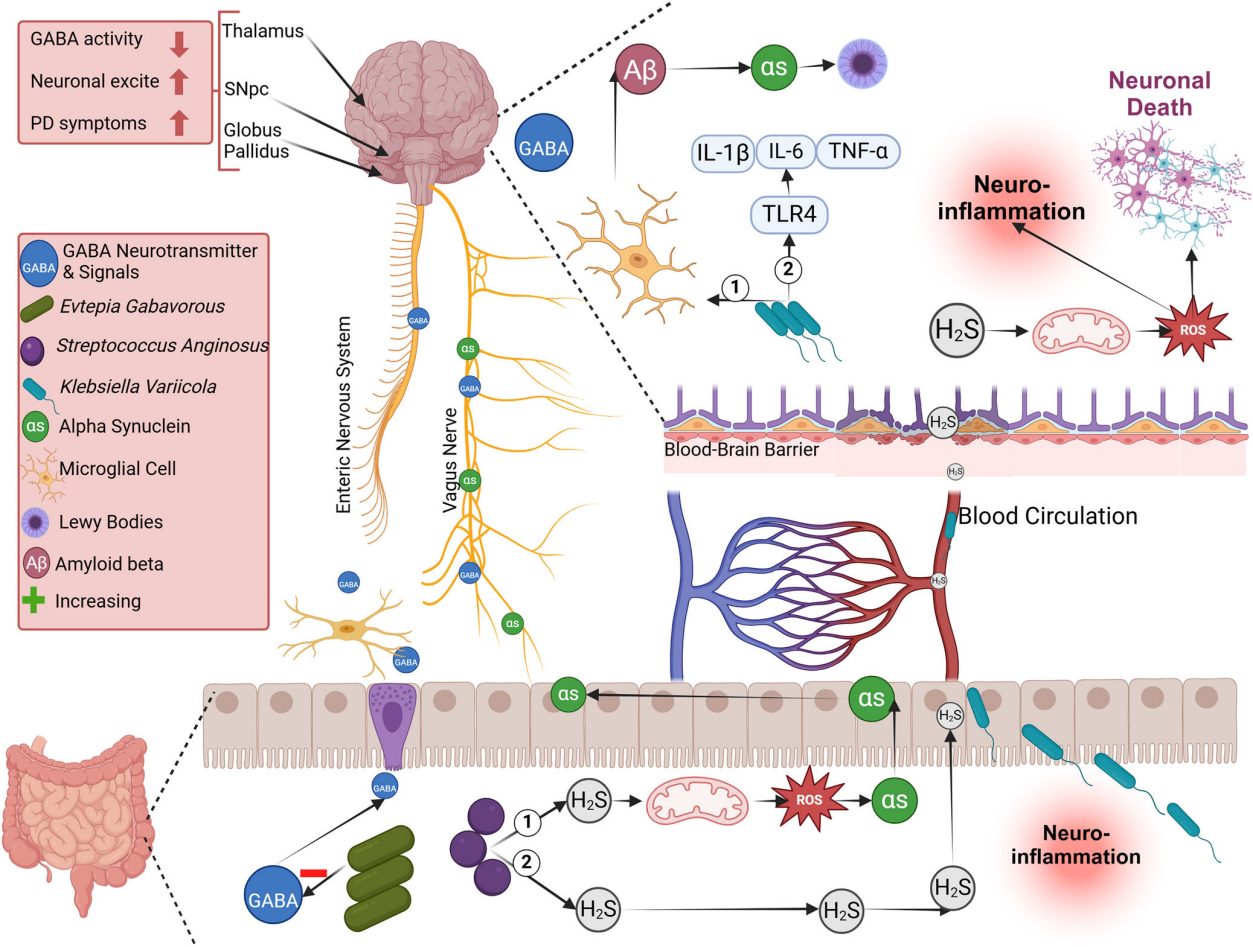
The complex interactions between gut microbiota and Parkinson’s Disease (PD) pathology. *Evtepia gabavorous* reduces gamma-aminobutyric acid (GABA) production by consuming it, impacting neuronal excitability in brain regions, thereby exacerbating Parkinson’s Disease (PD) symptoms. *Streptococcus anginosus* generates H_2_S, which disrupts mitochondrial functions, elevating ROS levels and encouraging alpha-synuclein (αSyn) aggregation and prion-like spread via the vagal nerve. Moreover*, Klebsiella variicola* targets microglial cells, augmenting beta-amyloid and αSyn production, potentially intensifying PD progression through increased pro-inflammatory responses mediated by toll-like receptors. Created with BioRender.com.

The intricate relationship between gut microbiota and the role it plays in the development of Parkinson's Disease (PD) has been an active research topic for the past couple of years. While considerable research has focused on elucidating this relationship, a consensus identification of the microbial species and their contributions remains unclear. From our meta-analysis of fifteen primary studies exploring this relationship, we have identified five homogeneous case-control studies of 16S rRNA V3-V4 regions comprising 1007 fecal samples that were selected for further analysis. This meta-analysis addresses the inconsistency of primary studies and other meta-analyses attributed to different regions and study designs, where we attempted to pinpoint the microbial players involved and their potential contributions to PD pathogenesis (Fig. 5).

In this work, we characterized a gut microbial composition consistently associated with PD across all of the included studies, a finding that is concordant with earlier research^20,23,31,33,39,40^. A significant increase in alpha diversity was observed in PD patients compared to Healthy Control (HC), which is indicative of a broader range of microbial species. Moreover, differences in beta diversity were identified in our analysis, indicating significantly distinct microbial community compositions consistently observed across other studies as well^20,21,23,31,41–44^. This was emphasized by notable bacterial shifts in PD patients compared to HC, collectively observed in other studies, where several genera showed an increase in abundance, namely Desulfovibrio, Enterococcus, Bifidobacterium, Hungatella, Lactobacillus, Parabacteroides, Akkermansia, and Alistipes^12,21,32,45–48^. Additionally, according to our knowledge, we report for the first time the abundance of the Weissella genus, which has been found to reduce pro-inflammatory markers^49^, and that of the Neglectibacter genus which has a fatty acid profile that may affect PD progression^50^.

Confirming previous findings, the involvement of the gut microbiome in the etiology of PD may involve alterations of short-chain fatty acids (SCFAs)-producing bacteria where some showed elevation and others depletion^12^. Some of these taxa namely, Bifidobacterium, Escherichia, Desulfovibrio, and Akkermansia, were elevated, potentially affecting intestinal barrier integrity and permeability^12,23,51^. Conversely, other genera such as Blautia, Faecalibacterium, Fusicatenibacter, Lachnospira, Roseburia, Prevotella, and Coprococcus were reduced, potentially affecting the gut health and reducing inflammation^23,45–47^. Furthermore, we report the abundance of a novel SCFA-producing genus in PD, namely Agathobaculum, which has been linked to gut health, reduced inflammation, and cholesterol levels, triglycerides, and TNF-α production^52,53^. Based on our results, we report the depletion of *Agathobaculum butyriciproducens,* which was found to have neuroprotective effects in a PD model^54^. Of interest, our functional analysis demonstrated that threonine, serine, and glycerolipid metabolism were enriched in PD samples, contributing to SCFAs production^55–57^. This is complemented by the enrichment of pathways involved in the degradation of branched-chain amino acids such as valine, leucine, and isoleucine, which can also be converted into branched-chain SCFAs^58^. This overall shift in microbial composition and SCFAs altered production suggests a potential link to PD progression, highlighting the need for further exploration of this area as that could serve as a biomarker or a therapeutic target for PD.

In addition to the microbial shift linked to PD and consequent alteration in SCFA, we have characterized several species likely to be implicated in the disease pathophysiology. *Klebsiella variicola*, a pro-inflammatory bacterium, emerged as one of the most distinctive and significantly enriched bacteria in PD patients across all five studies, with a relative abundance of 1.2%. *K. variicola* has been shown to significantly elevate the expression of pro-inflammatory cytokines, including TNF-α, IL-1β, and IL-6, as well as toll-like receptors (TLR2 and TLR9) in microglial cells^59,60^, likely by reaching the brain through blood circulation^59,61,62^. This elevation was reportedly correlated with a pro-inflammatory state in microglia, which subsequently enhances their phagocytic activity towards beta-amyloid (Aβ), facilitated by a 33-fold increase in the expression of the phagocytic receptor MSR1^59^. Accumulation of Aβ was suggested to promote the aggregation of α-synuclein (αsyn), which may exacerbate the pathology and progression of PD^63,64^. The survival and pathogenesis mechanisms of *K. variicola* were proposed to be linked to adaptive metabolism, biosynthesis pathways, stress resistance, and various virulence regulatory systems, such as the ABC transporter, and quorum sensing systems^65^. Next to *K.* *variicola*, the pro-inflammatory bacterium *E.* *siraeum* was also observed to be significantly abundant in PD patients. The inflammaging *E. siraeum* may disrupt gut barrier integrity and promote intestinal inflammation, which is linked to PD pathogenesis by exacerbating gut-brain axis dysfunction and contributing to disease progression^66^.

Our results also revealed elevated levels of hydrogen sulfide (H_2_S)-producing bacteria, such as *Streptococcus anginosus* and *Mordavella massiliensis*, in PD patients, which may result in excess H_2_S production and elevated reactive oxygen species (ROS) levels^67–72^. Increased ROS levels in the gut may promote α-syn aggregation into oligomers and fibrils, facilitating prion-like spreading from the gut to the brainstem and accelerating PD progression^73^. Moreover, H_2_S entering the bloodstream and reaching the brain might inhibit the cytochrome c oxidase enzyme—crucial for ATP generation in neurons—by binding to the CuB site and disrupting oxygen kinetics, ultimately impairing ATP production^70^, exacerbating neurotoxicity, and further advancing PD pathology^74^.

Our analysis further identified the novel gamma-aminobutyric acid (GABA) modulating species, *Evtepia gabavorous,* that is significantly more abundant in PD patients compared to HC. Strandwitz et al. identified *Evtepia gabavorous* as a GABA-eating bacterium that thrives in the presence of Bacteroides fragilis, a potent GABA producer^16^. Consistent with this, we observed an overall increase in the abundance of GABA-consuming species in PD samples relative to HC, reinforcing the role of GABA-modulating bacteria in PD. This aligns with emerging evidence linking gut dysbiosis to altered GABAergic signaling in PD. For instance, Barichella et al. (2021) observed dysregulation of microbial GABA metabolism pathways, particularly an increase in the GABA degradation pathway, in PD fecal metagenomes, suggesting a systemic imbalance in GABA homeostasis driven by gut microbiota^29^. The malfunction of the GABA pathway due to the increase of this GABA-eating bacteria contributes to mitochondrial dysfunction and oxidative stress, integral to PD’s pathology^75^. Reduced levels of GABA, which reach the brain through either enteric nerves or the vagus nerve, were suggested to be linked to the motor and non-motor symptoms in PD^76^. This could be explained by promoting mitochondrial dysfunction, oxidative stress, disruption of calcium homeostasis, increasing neuronal excitability, and contributing to dopaminergic neuron loss, which are highly related to PD neuropathology^77–80^. Notably, therapeutic interventions targeting microbial GABA metabolism show promise. Wu et al. (2023) demonstrated that supplementation with GABA-producing bacterial strains alleviates motor deficits such as tremors, likely by restoring GABAergic tone through gut-brain axis signaling^19^, and an illustrative figure for the role of the GABA is available in (Supplementary Fig. 5).

On the functional level, several PD-associated pathways were predicted to be significantly enriched in PD patients, including fatty acid metabolism, insulin resistance, RNA degradation, and the quorum sensing pathway. Fatty acid metabolism, a key pathway influencing PD pathophysiology, was found significantly enriched in PD patients, which was reported to affect neuronal differentiation and function^81–83^. Insulin resistance is another crucial pathway influencing PD by promoting αSyn aggregation, dopaminergic neuron loss, and heightened neuroinflammation, alongside mitochondrial dysfunction and disrupted autophagy due to PI3K/AKT/mTOR pathway hyperactivation, all of which exacerbate PD symptoms^84^. RNA degradation, particularly of circular RNAs, was also implicated in PD pathology. The degradation mediated by RNase L activates protein kinase R, linked to neuroinflammation and systemic responses in PD^85^.

Ultimately, this meta-analysis identified bacterial species that are significant in PD patients, pinpointing their exact roles in PD pathogenesis. Notably, *S. anginosus, K. variicola*, and *E. gabavorous* emerged as key species due to their potential involvement in mechanisms contributing to PD progression. This study represents the most robust meta-analysis conducted to date, offering novel insights into the relationship between the gut microbiome and PD at a deeper level. However, the inclusion of more studies with identical sequencing technology could further increase the power. To fully comprehend the complexity of these interactions and their clinical implications, further comprehensive research is essential. Future studies should include larger, more diverse study populations and incorporate transcriptomics and proteomics analyses to provide a deeper understanding of gut microbiota function. These aspects need to be considered and validated through additional cohort-based wet lab investigations. By expanding knowledge in this area, we can pave the way for more personalized and effective approaches for the early diagnosis of PD and novel therapeutic interventions.

## Methods

### Systematic search

A comprehensive search was initiated following the PRISMA (Preferred Reporting Items for Systematic Reviews and Meta-Analyses) guidelines on January 15, 2023, on the Sequence Read Archive (SRA) database^86^, which was selected as the primary source to identify any relevant research related to PD. This search employed specific keywords, including ‘Parkinson’ combined with at least one of the terms “metagenome,” “microbiome,” “amplicon sequence,” or ‘metagenomics,’ to identify 16S rRNA datasets. Studies included: (1) human participants, (2) case-control design comparing the gut microbiota of PD patients to HC, (3) 16S rRNA gene sequencing, (4) raw data is available, and (5) stool samples. Studies were excluded during the screening and eligibility stages if they: (1) lacked accompanying scientific papers, (2) utilized shotgun sequencing, (3) did not focus on V3-V4 regions of 16S rRNA, (4) lacked necessary metadata, or (5) included participants with recent antibiotic use.

### 16S rRNA gene data pre-processing

The raw 16S rRNA amplicon sequencing data in this study were obtained from the open-access database: SRA^86^, and initial processing was conducted using OCToPUS pipeline (v.1.0^87^). The reads were denoised using SPAdes (v.4.0.0^88^) and then merged using Mothur (v.1.48.1^89^), and then the contigs with ambiguities exceeding 8 homopolymers of the SILVA database (v.138.2^90^) were excluded. Additional denoising using IPED algorithm (v.1.0^91^) was performed, followed by de novo chimera removal by CATCh (v.1.0^92^). Operational taxonomic unit (OTU) clustering was conducted at a 97% identity using USEARCH (v.8.2.18^93^)) and classified against the Ribosomal Database Project dataset (v.16^94^). This approach enabled a more accurate and comprehensive understanding of microbial diversity. Quality control was conducted using the MetaQC module from the MetaOmics R package to ensure the reliability and consistency of the meta-analysis^38^. This includes Internal Quality Control (IQC), calculating the correlation of OTU pairs correlations across studies; Accuracy Quality Control (AQC), comparing the differentially abundant OTUs of each study to the remainder of the studies. Consistency Quality Control (CQC) examined the consistency of the ranking of differentially abundant OTUs from individual study analyses compared to the rest, thus evaluating the robustness of the results. The outcomes of these three quality control measures were then summarized using the Standardized Mean Rank (SMR), which provided a comprehensive indication of the studies’ overall level of consistency and reliability.

### Diversity analysis

Hypothesis testing for group differences in taxonomy and gene profile was conducted using the car package in R^95^ with *t.test* function to implement Mann-Whitney U test^96^ as Levene’s test with *leveneTest* indicated heterogeneous variances, and the Shapiro-Wilk test by *shapiro.test* function revealed non-normal data distribution. All statistically significant outcomes in this study were identified with p-values less than 0.05. Using the phyloseq R package^97^ alpha diversity indices were conducted by *plot_richness* function including the observed richness index (i.e., species richness, including rare species) and inverse simpson index (i.e., species richness and evenness^98^). To assess beta diversity, two different dimensionality reduction techniques were applied first using Rhea pipeline(v.1.1^99^), with calculations based on an unweighted UniFrac distance matrix. Analyses were performed separately for each study, and Ward’s hierarchical clustering along with NMDS plots were used for visualization. Secondly, Residuals and Covariance Modelling (RCM)^100^ was applied to account for inter-study differences. Additionally, accounting for cohort difference as a confounder was also applied for beta diversity calculations, which were further examined through PERMANOVA (Permutational Multivariate Analysis of Variance)^101^ using the vegan package’s *adonis2* function (v.2.6-4^102^) for both NMDS and RCM approaches.

### Biomarkers detection through meta-analysis

To identify taxa that differed significantly in abundance between groups PD and HC, Analysis of Composition of Microbiomes with Bias Correction (ANCOM-BC)^103^ was used, addressing challenges of compositionality, study difference, and sampling bias. For clearer representation, we constructed a cladogram showing phylogenetic relationships and abundance variations. MetaDE module of MetaOmics^38^ was then utilized performing a meta-analysis from the five studies with the random-effects model (REM)^104^. OTUs with false discovery rate (FDR) values below 0.05 were identified as significantly different, and the detected bacteria were classified using the NCBI Nucleotide BLAST, achieving approximately 97% identity and 100% coverage by rRNA/ITS database^105^. Another mapping was conducted to map the identified OTUs to the GABA-producing and GABA-consuming species presented in the work of Strandwitz et al.^16^; for this purpose, we applied BLAST with 100% coverage and at least 99% sequence identity. Then, to further assess the correlation between the GABA producers and consumers, in particular those that have been identified to be significant, we performed a linear model using the (linear model) *lm* function from the stats package^106^, coupled with Mann-Whitney U test^96^.

### Functional prediction

The metabolic and functional capabilities of the microbial communities were inferred through functional prediction using Tax4Fun function from themetagenomics package^107^. In addition, STAMP (Statistical Analysis of Metagenomic Profiles) was employed^108^ to conduct statistical hypothesis tests and generate visual comparisons of taxonomic and functional profiles between PD and HC. For this comparison, we applied Welch’s t-test, with alpha of 0.05 and an effect size of >0.1. To further explore the metabolic potential, the Kyoto Encyclopedia of Genes and Genomes (KEGG) database^109^ was used to determine the relevant pathways. Moreover, another pathway association analysis was conducted using MicrobiomeAnalyst^110^ to draw more connections between microbial functions.

### Machine learning

Machine learning techniques were employed to build a robust predictive model. The data used in this investigation were pre-processed using significant taxa across taxonomy level, and available metadata, namely the center name, city, and instrument. Prior to model training, categorical features were encoded with label encoding and one-hot encoding. A correlation matrix was computed, and features with a correlation > 0.1 were selected. The dataset was divided into two sets: training (80%), and testing (20%), with a random state of 50. Several machine learning models were compared, including Random Forest, XGBoost, and Decision tree^111–113^. The XGBoost classifier was selected due to its efficacy and robustness. Then it was initialized using grid search with the following hyperparameters: number of estimators set to 50, maximum depth at 3, column sample by tree and subsample at 0.6, use label encoder at False, and learning rate at 0.1. To avoid overfitting, the lasso regularization parameter set to 0.9 and the rigid regularization parameter set to 0.6.

## Supplementary information

Supplementary Information

## Data Availability

All data generated or analysed during this study are included in this published article [and its supplementary information files].

## Code availability

The underlying code for this study is not publicly available but may be made available to researchers on reasonable request from the corresponding author.
