# Supplementary material for: Proinflammatory and GABA eating bacteria in Parkinson's disease gut microbiome from a meta-analysis perspective
Source: NPJ Parkinsons Dis. 2025 Jun 3;11:145. doi: 10.1038/s41531-025-00950-z (PMC12134241; doi:10.1038/s41531-025-00950-z)
Supplement: Supplementary file 1 — Supplementary Information [file 41531_2025_950_MOESM1_ESM.pdf]

Supplementary Materials

Proinflammatory and GABA Eating Bacteria in Parkinson Disease Gut Microbiome From a Meta-analysis Prospective

Supplementary Figure 1: Microbial Difference Between Geographical Location Within Healthy Control (HC) Samples

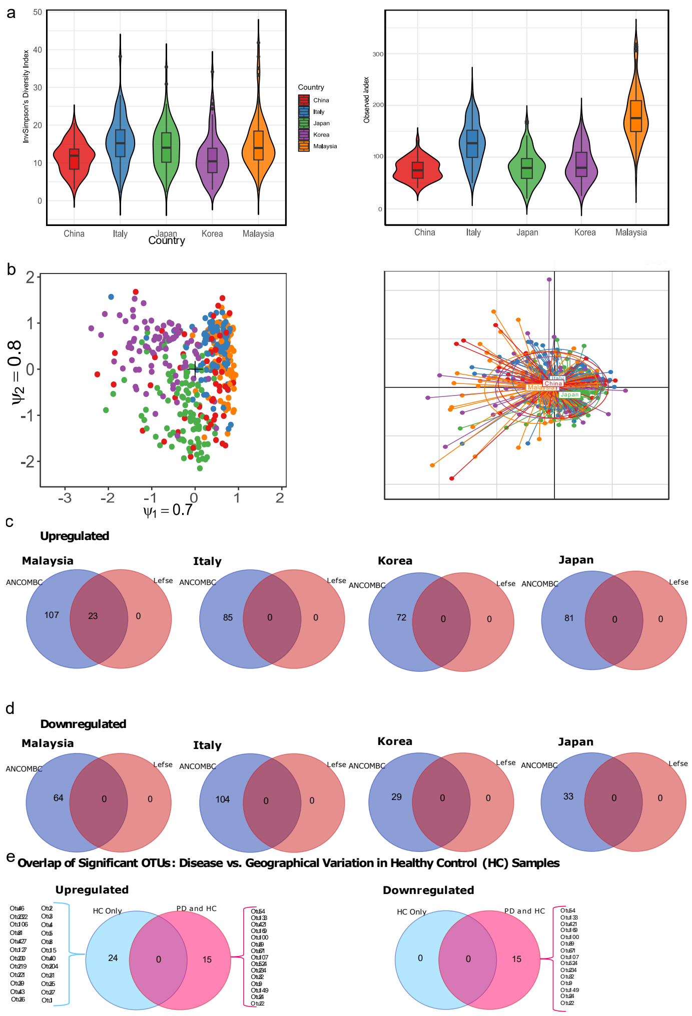

a Inverse Simpson richness Diversity Index and Observed Richness Index for species diversity within each study in Healthy Control (HC) samples. b Beta diversity analysis using Residual Variance–Covariance Modelling (RCM) and Rhea of microbial composition within geographical regions in HC samples. c Common upregulated significant species from ANCOM-BC and Lefse results across countries. d Common downregulated significant species from ANCOM-BC and Lefse results across countries. e Common significant species between the main meta-analysis and the HC-specific meta-analysis

Supplementary Figure 2: Microbial Difference Between Geographical Location Within Parkinson’s Disease (PD) Samples

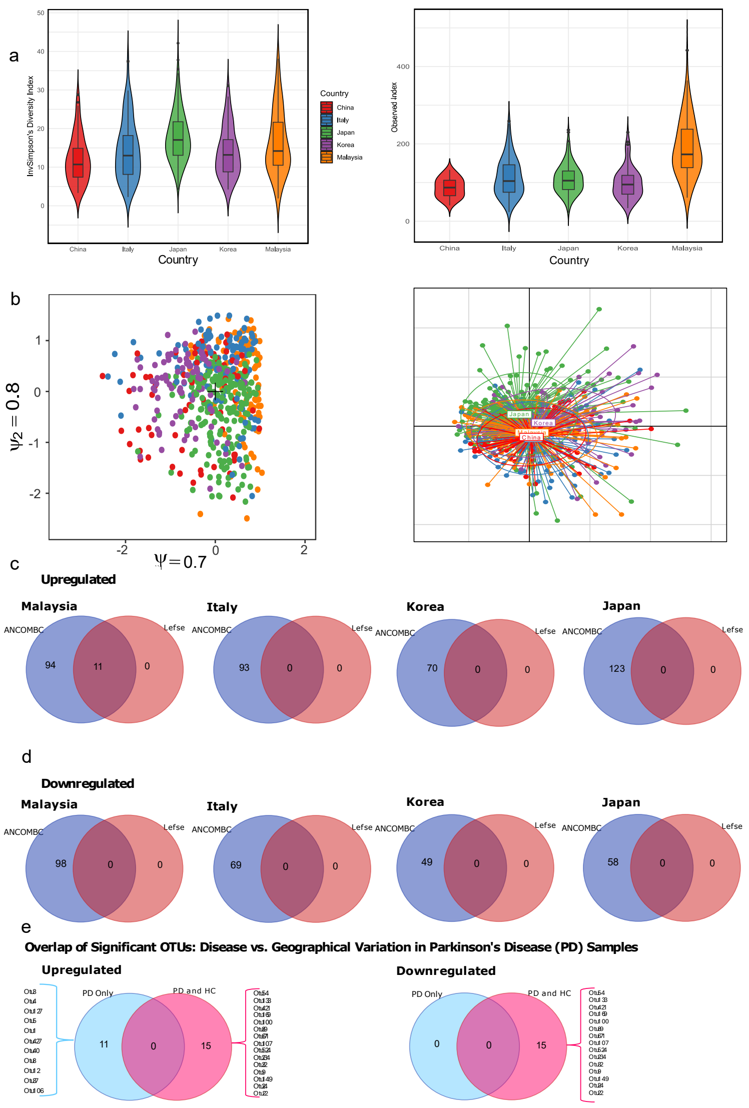

a Inverse Simpson Richness Diversity Index and Observed Richness Index measuring species diversity across studies in Parkinson’s Disease (PD) samples. b Beta diversity analysis with Residual Variance–Covariance Modelling (RCM) and Rhea, exploring microbial composition by geographical region in PD samples. c Shared upregulated significant species identified by ANCOM-BC and Lefse across countries. d Shared downregulated significant species detected by ANCOM-BC and Lefse across countries. e. Overlapping significant species between the main meta-analysis and the HC-specific meta-analysis.

Supplementary Figure 3: Residuals and Covariance Modelling Analysis Results

RCM for Country and Conditoin Classification

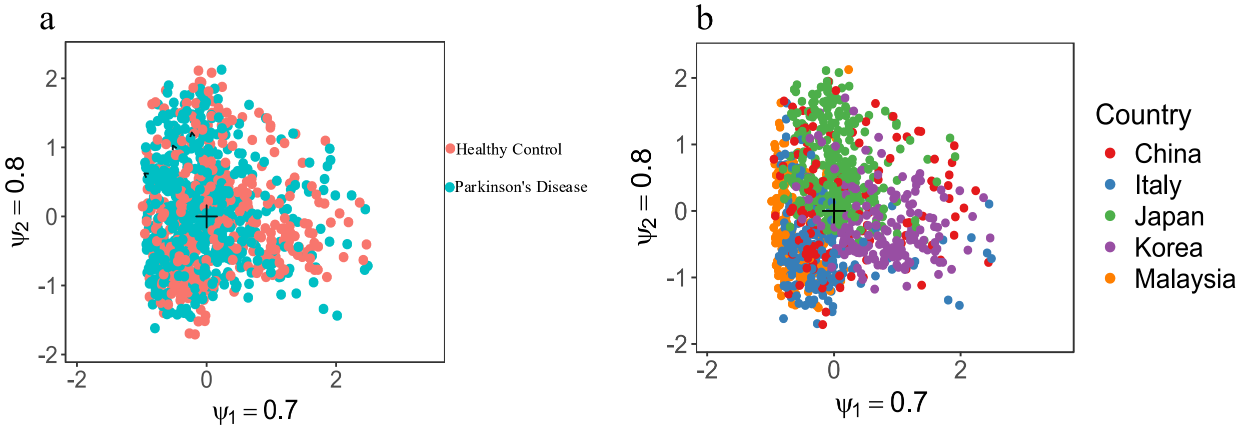

RCM for Country and Conditoin Classification with Confounder Effect

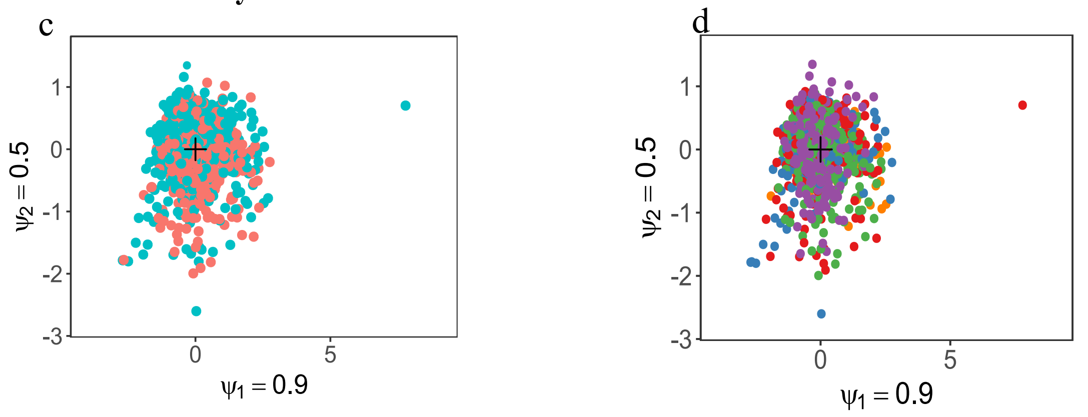

a Residuals and Covariance Modelling (RCM) plot for comparing between Parkinson's disease patients and healthy controls. **b** RCM plot comparing Parkinson’s Disease across different countries. **c** RCM plot for comparing between Parkinson's disease across different countries taking the geographical region as a confounder effect. **d** RCM plot for comparing Parkinson’s Disease Across Different Countries

Supplementary Figure 4: Linear Model Results for GABA-Eating and GABA-Producing Bacteria

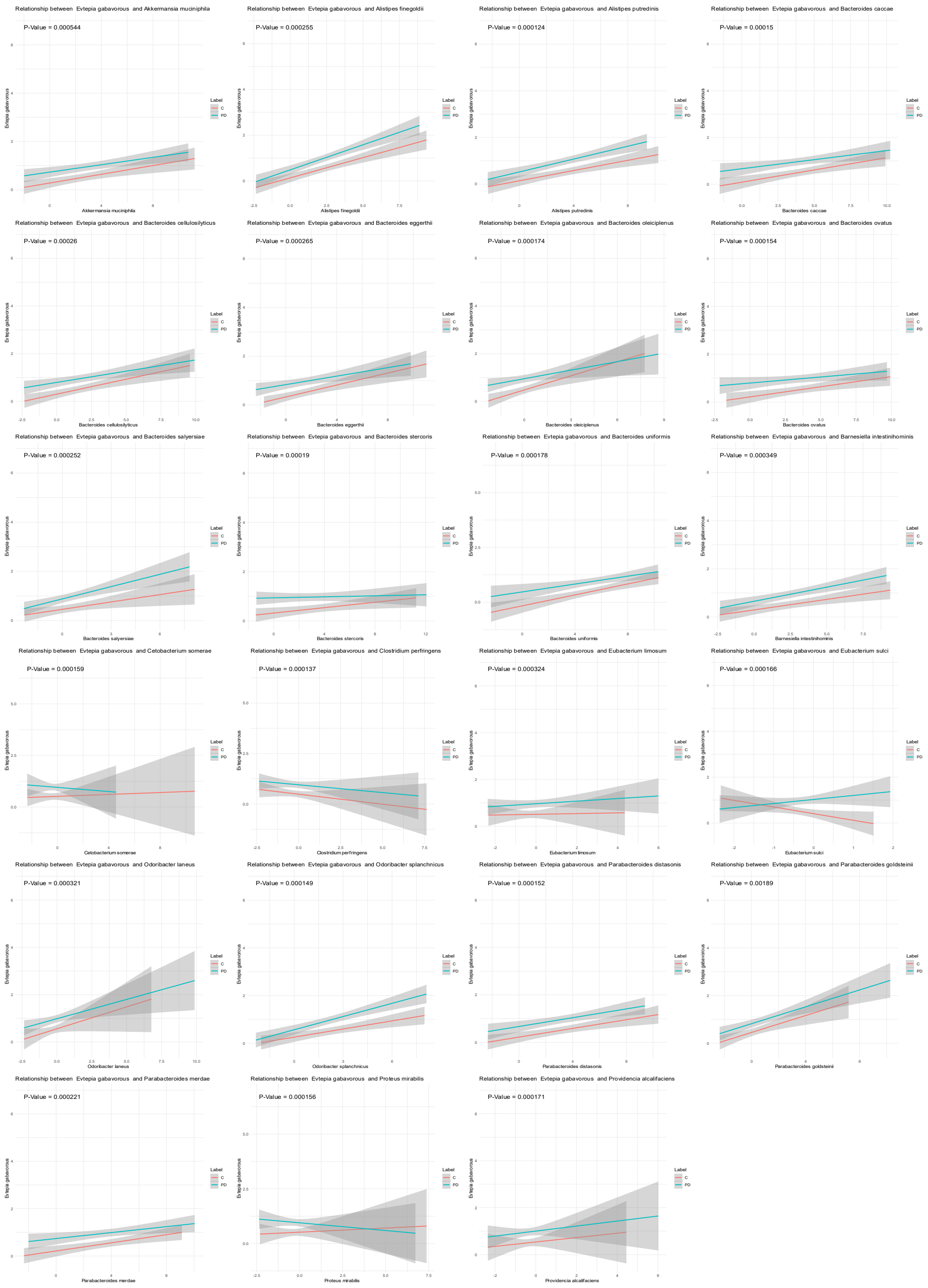

The results of all the linear models to identify the correlation between *Evetepia gabavorous* and all the GABA-producing bacterial species separately

Supplementary Figure 5: Gut-Brain Axis in Parkinson's Disease (PD): Role of GABA and Microbiome Dysbiosis

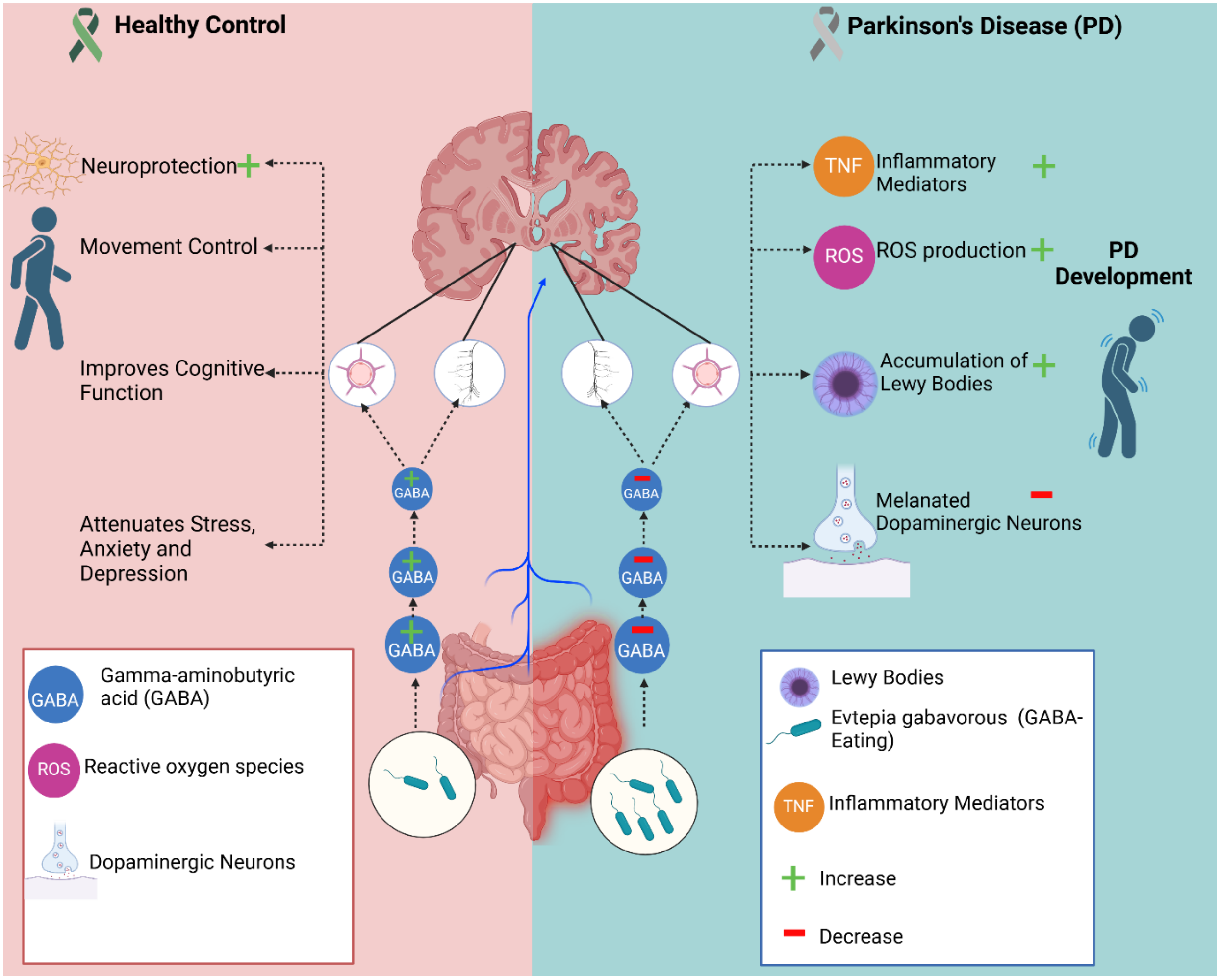

This illustration compares the gut-brain axis in healthy individuals versus Parkinson's disease (PD) patients. In a healthy state (left), Gamma-aminobutyric acid (GABA) produced in the gut supports neuroprotection, movement control, cognitive function, and emotional well-being. Conversely, in PD (right), reduced GABA levels, driven by the presence of Evtepia gabavorous (a GABA-eating bacteria), lead to increased inflammatory mediators and reactive oxygen species (ROS), contributing to Lewy body accumulation, loss of melanated dopaminergic neurons, and PD development. This disruption in the gut-brain communication pathway highlights the significant role of GABA-eating bacteria in neurodegenerative diseases. Created with BioRender.com.

Supplementary Table 1: Selection of Studies for Meta-Analysis Using PRISMA. Studies were included based on specific criteria, while exclusions were due to factors such as the use of animal models, lack of associated publications, absence of V3-V4 region coverage, unavailable metadata, or non-16S rRNA sequencing methods.

ID	Included/Excluded	Excluded
**PRJNA822998**	Included	_____
**PRJNA742875**	Included	_____
**PRJDB8639**	Included	_____
**PRJNA510730**	Included	_____
**PRJNA494620**	Included	_____
**PRJNA915969**	Excluded	animal model
**PRJNA914678**	Excluded	animal model
**PRJNA905333**	Excluded	animal model
**PRJEB56556**	Excluded	animal model
**PRJEB55464**	Excluded	animal model
**PRJNA895962**	Excluded	animal model
**PRJNA881840**	Excluded	no paper
**PRJNA852512**	Excluded	not covering v3-v4 region
**PRJEB52086**	Excluded	meta-data unavailable
**PRJNA838759**	Excluded	not 16s
**PRJNA834801**	Excluded	not 16s
**PRJDB12032**	Excluded	no paper
**PRJDB12031**	Excluded	no paper
**PRJNA822715**	Excluded	meta-data unavailable
**PRJNA821099**	Excluded	animal model
**PRJNA808166**	Excluded	not case-control
**PRJNA790457**	Excluded	not case-control
**PRJNA769968**	Excluded	not 16s
**PRJNA765486**	Excluded	animal model
**PRJNA764337**	Excluded	not case-control
**PRJNA762484**	Excluded	not 16s
**PRJNA756556**	Excluded	not 16s
**PRJNA753493**	Excluded	animal model
**PRJEB45549**	Excluded	not 16s
**PRJNA725395**	Excluded	animal model
**PRJNA720771**	Excluded	no paper
**PRJNA673724**	Excluded	animal model
**PRJNA662100**	Excluded	irrelevant topic
**PRJNA658581**	Excluded	animal model
**PRJEB32920**	Excluded	not case-control
**PRJEB32682**	Excluded	animal model

**PRJNA601994**	Excluded	not covering v3-v4 region
**PRJEB30615**	Excluded	not covering v3-v4 region
**PRJNA594156**	Excluded	antibiotic
**PRJNA588359**	Excluded	animal model
**PRJNA574851**	Excluded	animal model
**PRJNA564818**	Excluded	animal model
**PRJNA561023**	Excluded	meta-data unavailable
**PRJNA559904**	Excluded	animal model
**PRJEB27564**	Excluded	antibiotic
**PRJNA530401**	Excluded	no paper
**PRJEB30401**	Excluded	meta-data unavailable
**PRJNA454894**	Excluded	animal model
**PRJNA433459**	Excluded	not 16s
**PRJNA387564**	Excluded	irrelevant topic
**PRJEB14928**	Excluded	not covering v3-v4 region
**PRJNA381395**	Excluded	mentioned before
**PRJEB14674**	Excluded	not covering v3-v4 region
**PRJNA362482**	Excluded	irrelevant topic
**PRJEB17694**	Excluded	animal model
**PRJEB4927**	Excluded	454 GS FLX Titanium
**PRJNA268515**	Excluded	only one sample

Supplementary Table 2: Significant Taxonomic Levels. The microbial taxa enrichment between Parkinson's Disease (PD) samples and Healthy Control (HC) samples

Taxonomic Level	PD Enriched Taxa	HC Enriched Taxa
Phylum	Actinobacteria, Bacteria_unclassified, Synergistetes, Verrucomicrobia, Bacilli, Coriobacteriia, Firmicutes_unclassified, Synergistia, Verrucomicrobiae, Bifidobacteriales, Clostridia_unclassified, Eggerthellales, Lactobacillales, Synergistales.	Bacteroidetes, Alphaproteobacteria, Bacteroidia, Betaproteobacteria, Clostridia, and Negativicutes
Order	Bifidobacteriales, Clostridia_unclassified, Eggerthellales, Lactobacillales, Synergistales	Aeromonadales, Alphaproteobacteria_unclassified, Bacteroidales, Burkholderiales, Clostridiales, Pasteurellales, Selenomonadales.
Family	Akkermansiaceae, Bifidobacteriaceae, Clostridiales_Incertae_Sedis_XIII, Clostridiales_unclassified, Eggerthellaceae, Enterococcaceae, Lactobacillaceae, Synergistaceae.	Bacteroidaceae, Burkholderiales_unclassified, Lachnospiraceae, Pasteurellaceae, Prevotellaceae, Selenomonadaceae, Succinivibrionaceae, Sutterellaceae.
Genus	Bifidobacterium, Cloacibacillus, Clostridium_IV, Desulfovibrio, Eisenbergiella, Enterococcus, Hungatella, Intestinimonas, Lactobacillus, Ligilactobacillus, Limosilactobacillus, Longicatena, Merdimonas, Ruthenibacterium, Parabacteroides, Dysosmobacter, Streptococcus, Negativibacillus,	Acidaminococcaceae_unclassified, Agathobacter, Agathobaculum, Blautia, Clostridiaceae_1_unclassified, Coprococcus, Dialister, Dorea, Faecalibacterium, Fusicatenibacter, Gemmiger, Haemophilus, Lachnospira, Lachnospiraceae_unclassified,

	Flintibacter, Akkermansia, Alistipes, Eggerthella, Neglecta, Clostridiales_Incertae_Sedis_XIII_unclassified.	Lawsonibacter, Megamonas, Muribaculaceae_unclassified, Oscillibacter, Phocaeicola, Prevotella, Roseburia, Ruminococcus, Ruminococcus2, Succinivibrio, Terrisporobacter, Weissella, Anaerotignum, Ruminococcaceae_unclassified, Parasutterella.
Species	Bifidobacterium longum, Streptococcus anginosus, Lactobacillus acidophilus, Evtepia gabavorous, Bifidobacterium bifidum, Mordavella massiliensis, Eubacterium siraeum, Bifidobacterium pseudocatenulatum, Klebsiella variicola, Parabacteroides goldsteinii.	Lachnoclostridium edouardi, Clostridium fessum, Dialister invisus, Coprococcus eutactus, Agathobaculum butyriciproducens, Jutongia huaianensis, Haemophilus parainfluenzae, Clostridium porci, Lactobacillus rogosae.

Supplementary Table 3: Differential abundance analysis results of OTUs using MetaDE. The table presents the effect size and variance for each dataset, Z-values (zval), p-values (pval), and false discovery rate (FDR) for each OTU identified by MetaDE.

	ind.ES.Malaysia	ind.ES.Korea	ind.ES.Japan	ind.ES.Italy	ind.ES.China	ind.Var.Malaysia	ind.Var.Korea	ind.Var.Japan	ind.Var.Italy	ind.Var.China	zval	pval	FDR
Otu1	-0.2	-0	-0.1	-0.3	0.13	0.02	0.01	0.02	0.03	0.02	-1.2	0.2	0.35
Otu10	-0.7	0.12	-0.2	-0.3	-0.1	0.02	0.01	0.02	0.03	0.02	-1.7	0.07	0.18
Otu100	0.37	0.13	0.22	0.27	0.29	0.02	0.01	0.02	0.03	0.02	3.78	0	0
Otu102	-0.5	0	-0.2	0.09	0.24	0.02	0.01	0.02	0.03	0.02	-0.7	0.46	0.63
Otu104	0.08	-0.3	0.03	-0.1	0.01	0.02	0.01	0.02	0.03	0.02	-1	0.26	0.42
Otu106	-0.2	0.25	-0.1	-0.2	-0	0.02	0.01	0.02	0.03	0.02	-0.5	0.58	0.71
Otu107	0.08	0.25	0.17	0.25	0.35	0.02	0.01	0.02	0.03	0.02	3.36	0	0
Otu108	-0	0.04	-0	0	-0.1	0.02	0.01	0.02	0.03	0.02	-0.4	0.62	0.74
Otu109	-0	0.02	-0.1	0.6	0.49	0.02	0.01	0.02	0.03	0.02	1.24	0.18	0.32
Otu11	-0.3	0.29	-0.2	-0.3	-0.2	0.02	0.01	0.02	0.03	0.02	-1.2	0.18	0.32
Otu110	0.11	-0.2	0.1	0.31	0.07	0.02	0.01	0.02	0.03	0.02	0.65	0.47	0.64
Otu1112	-0	0.25	-0.1	0.13	0.14	0.02	0.01	0.02	0.03	0.02	1.5	0.1	0.25
Otu117	-1.2	0.06	0.15	0.31	0.12	0.02	0.01	0.02	0.03	0.02	-0.4	0.66	0.77
Otu12	0.09	0.19	-0	-0.2	0.47	0.02	0.01	0.02	0.03	0.02	1.05	0.25	0.41
Otu120	0.33	-0.1	-0	0.01	-0.1	0.02	0.01	0.02	0.03	0.02	0.25	0.78	0.88
Otu122	-0.4	0.01	-0.3	0	-0.1	0.02	0.01	0.02	0.03	0.02	-1.8	0.05	0.16
Otu1225	0.1	0.19	-0.2	0.23	0.3	0.02	0.01	0.02	0.03	0.02	1.37	0.14	0.29
Otu1228	-0.4	-0.3	-0.4	0.05	-0.1	0.02	0.01	0.02	0.03	0.02	-3	0	0.01
Otu124	-0.3	-0	-0.2	-0.4	0.05	0.02	0.01	0.02	0.03	0.02	-2	0.04	0.12
Otu125	-0	0.08	0.21	0.01	0.02	0.02	0.01	0.02	0.03	0.02	1	0.27	0.42
Otu1254	-0.3	-0.2	-0.4	0.25	-0.1	0.02	0.01	0.02	0.03	0.02	-1.4	0.12	0.27
Otu126	0.03	-0.2	-0	0.17	0.08	0.02	0.01	0.02	0.03	0.02	-0.4	0.67	0.77
Otu128	-0.3	0.1	-0.1	-0	0.19	0.02	0.01	0.02	0.03	0.02	-0.2	0.83	0.92
Otu129	0.67	0.21	0.15	0.22	-0	0.02	0.01	0.02	0.03	0.02	2.18	0.02	0.08
Otu13	-0.4	-0.2	-0.4	-0.3	-0.2	0.02	0.01	0.02	0.03	0.02	-4.7	1e-20	2.5625e-19
Otu130	-0.1	-0.4	-0.3	0.51	-0.4	0.02	0.01	0.02	0.03	0.02	-0.9	0.31	0.46
Otu131	0.19	0.14	-0.2	0.27	-0	0.02	0.01	0.02	0.03	0.02	0.98	0.28	0.43
Otu132	0	0.43	-0	0.22	0.52	0.02	0.01	0.02	0.03	0.02	2.07	0.03	0.1
Otu133	0.38	0.23	0.14	0.32	0.32	0.02	0.01	0.02	0.03	0.02	4.18	1.46341463415078e-05	0
Otu136	-0.3	-0.1	-0.3	0.07	0.1	0.02	0.01	0.02	0.03	0.02	-1.3	0.16	0.3

Otu14	-0	0.18	-0.2	-0.4	0.23	0.02	0.01	0.02	0.03	0.02	-0.2	0.86	0.92
Otu142	-0.4	-0.3	-0.2	0.08	-0.1	0.02	0.01	0.02	0.03	0.02	-2.8	0	0.02
Otu144	-0.2	-0	0.1	0.21	-0	0.02	0.01	0.02	0.03	0.02	-0.2	0.85	0.92
Otu1449	0.15	0.19	0.05	0.56	0.08	0.02	0.01	0.02	0.03	0.02	2.05	0.03	0.1
Otu145	-0.6	-0.3	-0.5	-0.4	-0.3	0.02	0.01	0.02	0.03	0.02	-5.8	1e-20	2.5625e-19
Otu1457	-0.5	0.06	-0.5	-0.2	-0.1	0.02	0.01	0.02	0.03	0.02	-2.3	0.01	0.06
Otu149	-0.1	0.42	0.26	0.16	0.43	0.02	0.01	0.02	0.03	0.02	2.68	0.01	0.03
Otu15	-0	-0	-0.3	-0.5	0.26	0.02	0.01	0.02	0.03	0.02	-0.8	0.39	0.55
Otu150	0.48	0.21	-0.2	0.24	-0.1	0.02	0.01	0.02	0.03	0.02	1.14	0.21	0.37
Otu152	-0.3	0.04	0.07	0.15	0.29	0.02	0.01	0.02	0.03	0.02	0.55	0.54	0.7
Otu156	-0.1	0.04	-0.1	-0.3	-0.2	0.02	0.01	0.02	0.03	0.02	-1.6	0.09	0.23
Otu157	0.4	0.13	-0.2	-0.1	0.44	0.02	0.01	0.02	0.03	0.02	1.02	0.26	0.42
Otu158	-0.1	-0.1	-0.1	-0.1	0	0.02	0.01	0.02	0.03	0.02	-1.4	0.13	0.27
Otu16	-0.4	-0.1	-0.3	0.12	-0	0.02	0.01	0.02	0.03	0.02	-1.7	0.08	0.2
Otu160	-0.1	-0.2	-0.4	0.09	-0.5	0.02	0.01	0.02	0.03	0.02	-2.2	0.02	0.07
Otu163	0.14	0.13	0.01	-0.1	-0.1	0.02	0.01	0.02	0.03	0.02	0.61	0.5	0.65
Otu165	-0.1	0.21	0.01	-0.4	0.3	0.02	0.01	0.02	0.03	0.02	0.09	0.92	0.96
Otu166	-0.2	0.06	-0.3	0.03	-0.1	0.02	0.01	0.02	0.03	0.02	-1.4	0.12	0.27
Otu168	0.14	0.12	0.13	0.22	-0	0.02	0.01	0.02	0.03	0.02	1.77	0.06	0.17
Otu169	0.4	0.35	0.23	0.06	0.11	0.02	0.01	0.02	0.03	0.02	3.91	5.85365853658093e-05	0
Otu17	-0.1	-0.2	-0	0.08	-0.1	0.02	0.01	0.02	0.03	0.02	-1.3	0.15	0.3
Otu170	-0.2	0.27	0.09	-0.1	0.26	0.02	0.01	0.02	0.03	0.02	0.66	0.47	0.64
Otu171	-0.3	-0.1	-0.2	-0.4	-0.2	0.02	0.01	0.02	0.03	0.02	-3.1	0	0.01
Otu1733	-0.2	-0	-0.2	-0	-0.1	0.02	0.01	0.02	0.03	0.02	-1.6	0.08	0.2
Otu175	0.22	0.29	0.09	0.16	0.16	0.02	0.01	0.02	0.03	0.02	3.05	0	0.01
Otu178	-0.1	0.14	-0.1	0.18	0.48	0.02	0.01	0.02	0.03	0.02	1.04	0.25	0.41
Otu182	-0.1	0.41	-0.3	0.11	0.34	0.02	0.01	0.02	0.03	0.02	0.85	0.35	0.51
Otu192	0.14	0.1	-0.2	0.06	-0.1	0.02	0.01	0.02	0.03	0.02	-0	0.97	0.98
Otu194	-0.2	0.17	0.14	0.16	0.38	0.02	0.01	0.02	0.03	0.02	1.36	0.14	0.29
Otu1949	-0.6	-0	-0.5	-0.2	-0.2	0.02	0.01	0.02	0.03	0.02	-2.7	0	0.03
Otu195	0.01	0.11	-0.3	0.18	0.19	0.02	0.01	0.02	0.03	0.02	0.44	0.62	0.74
Otu199	-0.2	-0	-0.2	0.4	-0.2	0.02	0.01	0.02	0.03	0.02	-0.5	0.59	0.72
Otu1991	-0.3	0.11	-0.3	-0.1	-0	0.02	0.01	0.02	0.03	0.02	-1.5	0.12	0.27
Otu2	-0.6	-0	-0	0.2	-0.4	0.02	0.01	0.02	0.03	0.02	-1.2	0.19	0.33

Otu20	-0.1	0.02	-0.2	-0.3	0.15	0.02	0.01	0.02	0.03	0.02	-0.9	0.34	0.5
Otu200	-0.2	0.2	0.16	0.16	0.18	0.02	0.01	0.02	0.03	0.02	1.68	0.07	0.19
Otu201	0.31	0.05	0.07	0	0.14	0.02	0.01	0.02	0.03	0.02	1.8	0.05	0.16
Otu2020	0.2	-0	0.1	-0.1	0.01	0.02	0.01	0.02	0.03	0.02	0.71	0.44	0.6
Otu204	-0.8	-0	-0.1	0.16	-0.5	0.02	0.01	0.02	0.03	0.02	-1.4	0.12	0.27
Otu2080	-0.1	-0	-0.3	0.15	-0.3	0.02	0.01	0.02	0.03	0.02	-1.5	0.09	0.23
Otu21	0.16	-0	-0.3	0.01	0.07	0.02	0.01	0.02	0.03	0.02	-0.4	0.68	0.78
Otu210	-0.5	-0.2	-0.4	-0.1	-0.2	0.02	0.01	0.02	0.03	0.02	-3.5	0	0
Otu214	0.02	0.53	-0.2	-0.2	0.41	0.02	0.01	0.02	0.03	0.02	0.65	0.48	0.64
Otu215	-0.3	0.22	-0.2	-0	-0.1	0.02	0.01	0.02	0.03	0.02	-1	0.29	0.43
Otu217	-0.6	-0.2	-0.5	-0.4	-0	0.02	0.01	0.02	0.03	0.02	-3.2	0	0.01
Otu219	-0.2	0.41	-0.4	0.23	0.32	0.02	0.01	0.02	0.03	0.02	0.45	0.62	0.74
Otu22	0.24	0.49	-0	0.02	0.37	0.02	0.01	0.02	0.03	0.02	2.43	0.01	0.05
Otu221	-0.1	-0.2	0.18	0.88	-0.1	0.02	0.01	0.02	0.04	0.02	0.62	0.5	0.65
Otu2220	0.48	0.26	0.27	0.18	0.32	0.02	0.01	0.02	0.03	0.02	4.73	1e-20	2.5625e-19
Otu225	-0	0.36	0.24	0.08	0.16	0.02	0.01	0.02	0.03	0.02	2.84	0	0.02
Otu229	0.13	0.43	-0.2	0	0.26	0.02	0.01	0.02	0.03	0.02	1.35	0.14	0.29
Otu23	-0.7	-0.1	-0.2	0.04	-0.2	0.02	0.01	0.02	0.03	0.02	-2	0.03	0.11
Otu230	-0.2	0.78	-0	-0.1	0.48	0.02	0.02	0.02	0.03	0.02	1.09	0.23	0.39
Otu232	0.16	0.16	-0.2	-0.1	0.09	0.02	0.01	0.02	0.03	0.02	0.57	0.53	0.69
Otu2322	-0.2	0.18	-0.2	-0.2	0.43	0.02	0.01	0.02	0.03	0.02	0.05	0.95	0.97
Otu233	0.19	0.2	-0.3	0.2	0.43	0.02	0.01	0.02	0.03	0.02	1.11	0.23	0.38
Otu234	0.2	0.2	0.17	0.01	0.4	0.02	0.01	0.02	0.03	0.02	3.18	0	0.01
Otu235	-0.5	-0.3	-0.4	0.04	-0.1	0.02	0.01	0.02	0.03	0.02	-2.8	0	0.02
Otu2366	0.08	0.4	0.1	-0.4	0.42	0.02	0.01	0.02	0.03	0.02	0.8	0.38	0.54
Otu24	0.27	0.06	0.28	0.21	0.06	0.02	0.01	0.02	0.03	0.02	2.59	0.01	0.03
Otu25	-0.4	-0.1	-0.1	-0.6	0.09	0.02	0.01	0.02	0.03	0.02	-1.7	0.06	0.18
Otu251	-0	0	-0.4	-0.1	0.08	0.02	0.01	0.02	0.03	0.02	-1	0.28	0.43
Otu26	0.03	0.1	0.21	-0.3	0.08	0.02	0.01	0.02	0.03	0.02	0.68	0.45	0.62
Otu263	0.28	0.19	-0.3	0.09	-0.1	0.02	0.01	0.02	0.03	0.02	0.24	0.79	0.88
Otu272	-0	0.18	-0.3	-0.1	-0	0.02	0.01	0.02	0.03	0.02	-0.4	0.64	0.75
Otu273	-0.5	-0.2	0.01	-0.1	-0.2	0.02	0.01	0.02	0.03	0.02	-2.3	0.02	0.06
Otu274	-0.2	-0.3	-0.5	0.06	-0.4	0.02	0.01	0.02	0.03	0.02	-3	0	0.01
Otu279	-0.2	-0	0.19	-0.1	-0.4	0.02	0.01	0.02	0.03	0.02	-1.1	0.23	0.38
Otu28	-0	-0	0.11	-0	-0.3	0.02	0.01	0.02	0.03	0.02	-0.5	0.57	0.71
Otu285	0.61	0.16	-0.1	0.2	-0.1	0.02	0.01	0.02	0.03	0.02	1.34	0.15	0.29

Otu286	-0.2	-0.3	-0.3	-0.2	-0	0.02	0.01	0.02	0.03	0.02	-3.3	0	0
Otu296	0.18	-0.2	0.16	0.03	0.18	0.02	0.01	0.02	0.03	0.02	0.85	0.35	0.51
Otu3	-0	0.09	0.1	0.06	0.24	0.02	0.01	0.02	0.03	0.02	1.39	0.13	0.28
Otu30	-0.1	-0	0.06	0.32	-0.1	0.02	0.01	0.02	0.03	0.02	0.08	0.93	0.96
Otu301	0.43	0.1	-0.1	-0	-0	0.02	0.01	0.02	0.03	0.02	1	0.27	0.42
Otu303	-0.2	-0	-0.3	-0.1	-0.4	0.02	0.01	0.02	0.03	0.02	-3	0	0.01
Otu305	0.12	0.19	-0.1	0.1	0.18	0.02	0.01	0.02	0.03	0.02	1.75	0.06	0.17
Otu31	-0.6	0.21	-0.3	0.15	-0	0.02	0.01	0.02	0.03	0.02	-0.8	0.38	0.54
Otu313	-0	0.29	0.23	0.1	0.15	0.02	0.01	0.02	0.03	0.02	2.56	0.01	0.04
Otu314	0.19	0.11	-0.1	-0.1	-0.1	0.02	0.01	0.02	0.03	0.02	0.23	0.8	0.88
Otu32	0.2	0.14	0.09	0.5	0.24	0.02	0.01	0.02	0.03	0.02	2.93	0	0.02
Otu327	-0	0.43	-0.2	0.25	0.49	0.02	0.01	0.02	0.03	0.02	1.3	0.16	0.3
Otu33	0.1	0.27	-0	0.38	0.23	0.02	0.01	0.02	0.03	0.02	2.67	0.01	0.03
Otu333	0.24	0.5	-0.1	0.09	0.22	0.02	0.01	0.02	0.03	0.02	1.98	0.03	0.12
Otu334	-0.4	0.39	0.04	-0.1	0.06	0.02	0.01	0.02	0.03	0.02	-0	0.99	0.99
Otu337	0.32	0.11	-0.3	0.33	-0.1	0.02	0.01	0.02	0.03	0.02	0.52	0.57	0.71
Otu338	0.22	0.31	0.37	-0.2	0.22	0.02	0.01	0.02	0.03	0.02	2.4	0.01	0.05
Otu340	0.31	0.41	0.27	0.37	0.07	0.02	0.01	0.02	0.03	0.02	4.55	1e-20	2.5625e-19
Otu35	-0.3	0.19	-0.5	0.08	-0.2	0.02	0.01	0.02	0.03	0.02	-1.1	0.22	0.37
Otu367	0.01	0.19	-0.2	-0.2	0.26	0.02	0.01	0.02	0.03	0.02	0.17	0.84	0.92
Otu368	0.16	0.27	-0.2	0.02	-0	0.02	0.01	0.02	0.03	0.02	0.91	0.32	0.47
Otu369	0.27	0.04	0.09	0.09	0.06	0.02	0.01	0.02	0.03	0.02	1.65	0.08	0.2
Otu37	-0.2	0.24	-0.1	-0.3	0.29	0.02	0.01	0.02	0.03	0.02	-0.1	0.93	0.96
Otu371	0.18	0.04	-0	0.15	0	0.02	0.01	0.02	0.03	0.02	0.98	0.28	0.43
Otu376	0.2	0.09	0.08	0.11	0.12	0.02	0.01	0.02	0.03	0.02	1.81	0.05	0.16
Otu38	0.04	0.09	0.02	-0.1	0.07	0.02	0.01	0.02	0.03	0.02	0.53	0.56	0.7
Otu388	0.27	0.07	-0.1	0.05	0.15	0.02	0.01	0.02	0.03	0.02	1.42	0.12	0.27
Otu39	-0	0.22	-0.1	-0.3	0.29	0.02	0.01	0.02	0.03	0.02	0.3	0.74	0.84
Otu390	-0.2	-0.1	-0.1	0.15	0.05	0.02	0.01	0.02	0.03	0.02	-1.1	0.21	0.37
Otu391	-0	-0	-0.1	0.05	0.23	0.02	0.01	0.02	0.03	0.02	0.32	0.72	0.82
Otu4	-0.1	0.62	0.12	-0.1	0.24	0.02	0.02	0.02	0.03	0.02	1.23	0.18	0.32
Otu40	0.02	0.28	-0.2	-0.2	0.15	0.02	0.01	0.02	0.03	0.02	0.52	0.57	0.71
Otu414	-0	0.03	-0.1	-0.1	-0.3	0.02	0.01	0.02	0.03	0.02	-1.3	0.17	0.31
Otu42	-0.7	-0.4	-0.3	-0.3	-0.1	0.02	0.01	0.02	0.03	0.02	-3.7	0	0
Otu421	0.38	0.35	0.1	0.55	0.19	0.02	0.01	0.02	0.03	0.02	3.92	5.36585365853437e-05	0
Otu423	-0.4	-0	-0.3	0.09	-0	0.02	0.01	0.02	0.03	0.02	-1.6	0.09	0.22
Otu427	-0.4	0.17	-0.2	-0.3	0.3	0.02	0.01	0.02	0.03	0.02	-0.6	0.48	0.64

Otu428	-0.2	0.03	-0.2	0.16	0.15	0.02	0.01	0.02	0.03	0.02	-0.3	0.75	0.84
Otu43	-0.4	-0.2	-0.2	-0.1	0.06	0.02	0.01	0.02	0.03	0.02	-2.3	0.01	0.06
Otu44	0.48	0.64	0.21	0.48	0.16	0.02	0.02	0.02	0.03	0.02	4.5	1e-20	2.5625e-19
Otu45	-0.5	-0.1	-0.2	-0.1	0.07	0.02	0.01	0.02	0.03	0.02	-1.8	0.05	0.16
Otu46	-0.5	0.2	-0.1	-0	0.19	0.02	0.01	0.02	0.03	0.02	-0.4	0.64	0.75
Otu462	0.01	0.21	0.01	0.3	0.33	0.02	0.01	0.02	0.03	0.02	2.39	0.01	0.05
Otu467	0.13	-0.2	-0.1	0.3	0.07	0.02	0.01	0.02	0.03	0.02	0.09	0.92	0.96
Otu47	-0.1	-0.2	0.11	0.13	-0.1	0.02	0.01	0.02	0.03	0.02	-0.5	0.59	0.72
Otu471	0.23	0.15	-0	0.34	0.25	0.02	0.01	0.02	0.03	0.02	2.67	0.01	0.03
Otu478	-0	0.15	-0.3	0.07	0.35	0.02	0.01	0.02	0.03	0.02	0.47	0.6	0.72
Otu48	0.05	0.51	0.08	-0.3	0.5	0.02	0.01	0.02	0.03	0.02	1.3	0.16	0.3
Otu481	0.21	-0	0.11	-0.3	-0	0.02	0.01	0.02	0.03	0.02	0.12	0.89	0.96
Otu493	0.04	0.2	-0	-0.2	-0	0.02	0.01	0.02	0.03	0.02	0.48	0.6	0.72
Otu496	-0.1	-0.3	-0.2	0.41	0.2	0.02	0.01	0.02	0.03	0.02	-0.1	0.91	0.96
Otu497	0.34	0.41	-0.1	0.19	0.43	0.02	0.01	0.02	0.03	0.02	2.33	0.01	0.06
Otu498	-0.1	0.56	-0.1	0.22	0.14	0.02	0.01	0.02	0.03	0.02	1.34	0.15	0.29
Otu5	0.21	0.02	0.35	-0.1	0.33	0.02	0.01	0.02	0.03	0.02	2.03	0.03	0.11
Otu50	0.13	0.08	-0	0.05	-0.1	0.02	0.01	0.02	0.03	0.02	0.44	0.63	0.74
Otu516	0.03	0.25	0.13	0.13	0.17	0.02	0.01	0.02	0.03	0.02	2.34	0.01	0.06
Otu524	0.5	0.63	-0	0.48	0.27	0.02	0.02	0.02	0.03	0.02	3.26	0	0.01
Otu539	0.14	0.28	0.16	0.34	-0.3	0.02	0.01	0.02	0.03	0.02	1.31	0.15	0.3
Otu54	0.43	0.26	0.16	0.16	0.54	0.02	0.01	0.02	0.03	0.02	4.2	1.46341463415078e-05	0
Otu55	-0	-0.4	-0.3	-0.1	-0.5	0.02	0.01	0.02	0.03	0.02	-2.9	0	0.02
Otu555	0.05	-0.2	-0.3	0.17	-0.3	0.02	0.01	0.02	0.03	0.02	-1.3	0.15	0.29
Otu556	0.05	0.17	0	-0	0.29	0.02	0.01	0.02	0.03	0.02	1.75	0.06	0.17
Otu56	-0.1	0.36	-0.1	0.11	0.36	0.02	0.01	0.02	0.03	0.02	1.04	0.25	0.41
Otu562	0.3	-0	-0	0.3	0.03	0.02	0.01	0.02	0.03	0.02	1.37	0.14	0.29
Otu58	-0.2	-0.2	-0.5	0.04	0.08	0.02	0.01	0.02	0.03	0.02	-1.6	0.08	0.21
Otu6	-1	-0	-0.1	-0.4	-0.1	0.02	0.01	0.02	0.03	0.02	-1.8	0.06	0.17
Otu61	-0.1	0.08	0.16	0.11	0.25	0.02	0.01	0.02	0.03	0.02	1.41	0.13	0.27
Otu610	-0.1	0.04	-0.2	-0.4	0.05	0.02	0.01	0.02	0.03	0.02	-1.4	0.13	0.27
Otu62	0.13	0.06	0.05	0.11	0.44	0.02	0.01	0.02	0.03	0.02	2.09	0.03	0.1
Otu625	-0.6	0.08	-0.2	-0.2	-0.2	0.02	0.01	0.02	0.03	0.02	-2	0.03	0.11
Otu63	0.03	0.07	-0.2	0.01	0.17	0.02	0.01	0.02	0.03	0.02	0.17	0.85	0.92
Otu638	0.15	0.23	-0.1	0.32	-0	0.02	0.01	0.02	0.03	0.02	1.47	0.11	0.26
Otu64	-0.8	0.11	-0.1	0.07	0.09	0.02	0.01	0.02	0.03	0.02	-0.7	0.42	0.59
Otu67	-0.5	-0.2	-0.1	-0	-0.1	0.02	0.01	0.02	0.03	0.02	-2.1	0.02	0.09

Otu671	0.26	0.25	0.16	0.32	0.15	0.02	0.01	0.02	0.03	0.02	3.48	0	0
Otu68	-0	0.33	-0.3	0.27	0.61	0.02	0.01	0.02	0.03	0.02	1.24	0.18	0.32
Otu69	-0.2	0.08	-0.2	-0.1	0.09	0.02	0.01	0.02	0.03	0.02	-1	0.29	0.43
Otu7	-0.7	-0.7	-0.7	-0.2	-0.6	0.02	0.02	0.02	0.03	0.02	-5.5	1e-20	2.5625e-19
Otu70	0.03	0.28	-0.2	-0.3	0.53	0.02	0.01	0.02	0.03	0.02	0.54	0.55	0.7
Otu71	-0.4	0.05	0.11	-0	0.2	0.02	0.01	0.02	0.03	0.02	-0.1	0.94	0.96
Otu719	-0.4	-0.1	-0.6	0.1	-0.1	0.02	0.01	0.02	0.03	0.02	-1.8	0.06	0.16
Otu72	-0.6	0.03	-0.4	-0.1	-0.3	0.02	0.01	0.02	0.03	0.02	-2.5	0.01	0.04
Otu724	-0.1	0.16	-0.2	0.04	-0	0.02	0.01	0.02	0.03	0.02	-0.1	0.93	0.96
Otu76	0.07	-0.3	0.14	0.23	-0.1	0.02	0.01	0.02	0.03	0.02	-0	0.98	0.98
Otu764	-0.2	0.14	-0.1	0.01	-0.1	0.02	0.01	0.02	0.03	0.02	-0.6	0.49	0.64
Otu78	-0.6	-0.2	-0.6	0.17	-0.4	0.02	0.01	0.02	0.03	0.02	-2.4	0.01	0.05
Otu79	-0.2	0.32	0.22	-0.1	0.53	0.02	0.01	0.02	0.03	0.02	1.06	0.25	0.41
Otu8	-0	-0	-0.2	-0.1	0.31	0.02	0.01	0.02	0.03	0.02	-0.1	0.9	0.96
Otu81	-0.1	0.23	-0.1	0.06	0.42	0.02	0.01	0.02	0.03	0.02	0.89	0.33	0.48
Otu82	0.22	0.25	-0	0.24	-0	0.02	0.01	0.02	0.03	0.02	2.04	0.03	0.11
Otu826	0.31	-0.1	0.06	-0.2	-0	0.02	0.01	0.02	0.03	0.02	0.11	0.9	0.96
Otu83	0.07	0.05	0.19	-0	0.25	0.02	0.01	0.02	0.03	0.02	1.63	0.08	0.2
Otu85	0.28	0.1	0.16	0.29	0.36	0.02	0.01	0.02	0.03	0.02	3.38	0	0
Otu86	-0.3	-0.2	-0.6	-0.1	-0.5	0.02	0.01	0.02	0.03	0.02	-3.7	0	0
Otu87	-0.5	-0.3	-0.4	0.31	-0.2	0.02	0.01	0.02	0.03	0.02	-1.5	0.1	0.23
Otu89	0.48	0.21	0.35	0.02	0.28	0.02	0.01	0.02	0.03	0.02	3.77	0	0
Otu9	0.17	0.08	0.17	0.24	0.36	0.02	0.01	0.02	0.03	0.02	2.91	0	0.02
Otu92	-0.7	-0.2	-0.3	0.23	0.03	0.02	0.01	0.02	0.03	0.02	-1.2	0.21	0.36
Otu93	-0.1	-0.1	0.15	0.13	0.37	0.02	0.01	0.02	0.03	0.02	1.01	0.27	0.42
Otu932	0.03	0.14	-0.2	-0.1	0.08	0.02	0.01	0.02	0.03	0.02	-0	0.96	0.98
Otu94	0.45	0.49	0.21	0.24	0.35	0.02	0.01	0.02	0.03	0.02	5.69	1e-20	2.5625e-19
Otu949	0.06	0.13	-0.1	0.14	0.31	0.02	0.01	0.02	0.03	0.02	1.61	0.08	0.21
Otu97	0.37	0.26	0.31	0.26	0.63	0.02	0.01	0.02	0.03	0.02	5.36	1e-20	2.5625e-19
Otu98	-0.2	-0	-0	-0.1	0.13	0.02	0.01	0.02	0.03	0.02	-0.6	0.5	0.65
Otu99	0.05	0.13	0.02	0.06	0.38	0.02	0.01	0.02	0.03	0.02	1.97	0.04	0.12

Supplementary Table 4: Functional Pathway Analysis Results Using MicrobiomeAnalyst. Identified metabolic pathways based on MicrobiomeAnalyst listing the pathways along with their size, observed hits, statistical scores, and multiple testing corrections (Holm and false discovery rate (FDR)).

Pathway	Size	Hits	Statistic Q	Expected Q	Pval	Holm p	FDR
**Glycerolipid metabolism**	17	17	0.943613	0.099404	1.43E-05	0.001447	0.000104
**Toluene degradation**	9	9	0.870863	0.099404	1.57E-05	0.001574	0.000107
**Tryptophan metabolism**	24	24	0.731475	0.099404	1.84E-05	0.001821	0.00011
**Ascorbate and aldarate metabolism**	23	23	0.728021	0.099404	1.90E-05	0.001859	0.00011
**Valine, leucine and isoleucine degradation**	23	23	0.80172	0.099404	1.92E-05	0.001866	0.00011
**Glycine, serine and threonine metabolism**	40	40	0.721692	0.099404	2.07E-05	0.001988	0.00011
**Biosynthesis of type II polyketide products**	3	3	1.589317	0.099404	2.08E-05	0.001988	0.00011
**Carotenoid biosynthesis**	10	10	0.929013	0.099404	2.56E-05	0.002404	0.000129
**Sulfur metabolism**	14	14	1.00956	0.099404	2.87E-05	0.002666	0.000139
**Ubiquinone and other terpenoid-quinone biosynthesis**	23	23	0.673829	0.099404	3.34E-05	0.003072	0.000149
**Purine metabolism**	42	42	0.773646	0.099404	3.35E-05	0.003072	0.000149
**Cysteine and methionine metabolism**	36	36	0.780241	0.099404	4.21E-05	0.003787	0.000181
**Propanoate metabolism**	34	34	0.749184	0.099404	4.93E-05	0.004388	0.000204
**Lipoic acid metabolism**	10	10	0.875764	0.099404	5.84E-05	0.005137	0.000227
**Tyrosine metabolism**	28	28	0.662626	0.099404	5.86E-05	0.005137	0.000227
**Valine, leucine and isoleucine biosynthesis**	4	4	0.807676	0.099404	7.70E-05	0.006619	0.000288
**Glutathione metabolism**	15	15	0.846876	0.099404	8.17E-05	0.006947	0.000294
**Caffeine metabolism**	2	2	0.962297	0.099404	8.36E-05	0.007019	0.000294
**Pentose phosphate pathway**	32	32	0.743873	0.099404	9.04E-05	0.007505	0.000308
**Butanoate metabolism**	31	31	0.723309	0.099404	0.000105	0.008646	0.000349
**Lysine biosynthesis**	19	19	0.699933	0.099404	0.000117	0.009487	0.000377
**alpha-Linolenic acid metabolism**	3	3	0.938851	0.099404	0.000127	0.010122	0.000397
**Fatty acid degradation**	13	13	1.060535	0.099404	0.000137	0.010857	0.000418
**Phenylalanine metabolism**	29	29	0.639678	0.099404	0.00014	0.010957	0.000418
**Starch and sucrose metabolism**	41	41	0.682611	0.099404	0.000159	0.012274	0.000448
**Riboflavin metabolism**	17	17	0.587332	0.099404	0.000161	0.012274	0.000448
**Caprolactam degradation**	6	6	0.932922	0.099404	0.000162	0.012274	0.000448
**Inositol phosphate metabolism**	14	14	0.644602	0.099404	0.000185	0.013702	0.000494
**Carbon fixation pathways in prokaryotes**	25	25	0.656982	0.099404	0.000187	0.013702	0.000494
**Pyrimidine metabolism**	28	28	0.731449	0.099404	0.000197	0.014156	0.000507
**Biosynthesis of enediyne antibiotics**	2	2	1.139609	0.099404	0.000201	0.014302	0.000508
**Taurine and hypotaurine metabolism**	8	8	0.551992	0.099404	0.000217	0.01521	0.000536
**Cyanoamino acid metabolism**	6	6	0.946689	0.099404	0.000249	0.017169	0.000601
**Chloroalkane and chloroalkene degradation**	8	8	0.628773	0.099404	0.000296	0.020153	0.000702
**Chlorocyclohexane and chlorobenzene degradation**	13	13	0.60652	0.099404	0.000351	0.023529	0.000813

**Histidine metabolism**	15	15	0.657097	0.099404	0.000357	0.023587	0.000813
**Pinene, camphor and geraniol degradation**	4	4	0.889578	0.099404	0.000366	0.023816	0.000817
**D-Amino acid metabolism**	13	13	0.569777	0.099404	0.000392	0.025067	0.000857
**Steroid hormone biosynthesis**	2	2	0.866929	0.099404	0.000478	0.030113	0.001027
**Pentose and glucuronate interconversions**	36	36	0.637963	0.099404	0.000521	0.032289	0.001098
**Isoquinoline alkaloid biosynthesis**	6	6	0.545307	0.099404	0.000587	0.035779	0.001215
**Arginine biosynthesis**	18	18	0.560083	0.099404	0.0008	0.047978	0.001609
**Porphyrin metabolism**	44	44	0.480768	0.099404	0.000814	0.048013	0.001609
**N-Glycan biosynthesis**	5	5	0.571708	0.099404	0.000818	0.048013	0.001609
**Retinol metabolism**	2	2	1.071142	0.099404	0.000843	0.04804	0.001616
**Staurosporine biosynthesis**	2	2	0.710369	0.099404	0.000863	0.048347	0.001616
**Drug metabolism - other enzymes**	11	11	0.718762	0.099404	0.000864	0.048347	0.001616
**Teichoic acid biosynthesis**	2	2	1.056766	0.099404	0.000969	0.052347	0.001759
**Galactose metabolism**	25	25	0.576553	0.099404	0.00097	0.052347	0.001759
**beta-Alanine metabolism**	20	20	0.506424	0.099404	0.001001	0.052347	0.001775
**Aminobenzoate degradation**	13	13	0.486854	0.099404	0.00101	0.052347	0.001775
**Fluorobenzoate degradation**	9	9	0.449945	0.099404	0.001097	0.054875	0.0019
**Naphthalene degradation**	4	4	0.826527	0.099404	0.00131	0.064175	0.002234
**Dioxin degradation**	9	9	0.605258	0.099404	0.001399	0.067166	0.002352
**Tetracycline biosynthesis**	2	2	0.706279	0.099404	0.001501	0.070536	0.002487
**Phosphonate and phosphinate metabolism**	9	9	0.715787	0.099404	0.001639	0.075398	0.002678
**Lysine degradation**	16	16	0.597423	0.099404	0.001918	0.086291	0.003089
**Pyruvate metabolism**	34	34	0.533076	0.099404	0.002051	0.090256	0.00326
**Nitrogen metabolism**	15	15	0.434275	0.099404	0.002392	0.102877	0.00375
**Amino sugar and nucleotide sugar metabolism**	61	61	0.463424	0.099404	0.002728	0.114562	0.004219
**Photosynthesis**	4	4	0.447671	0.099404	0.003332	0.136628	0.005086
**Alanine, aspartate and glutamate metabolism**	17	17	0.539743	0.099404	0.004144	0.165764	0.006243
**Fructose and mannose metabolism**	40	40	0.397106	0.099404	0.004424	0.172536	0.006579
**Tropane, piperidine and pyridine alkaloid biosynthesis**	5	5	0.422386	0.099404	0.005255	0.199694	0.007716
**O-Antigen nucleotide sugar biosynthesis**	29	29	0.421712	0.099404	0.005382	0.199694	0.007804
**Citrate cycle (TCA cycle)**	14	14	0.544199	0.099404	0.005817	0.209407	0.00833
**Ether lipid metabolism**	4	4	0.561241	0.099404	0.006016	0.210554	0.00851
**Nitrotoluene degradation**	4	4	0.456333	0.099404	0.006131	0.210554	0.008568
**Biosynthesis of various antibiotics**	4	4	0.499683	0.099404	0.006538	0.215755	0.008925
**Selenocompound metabolism**	8	8	0.620421	0.099404	0.00654	0.215755	0.008925
**Carbon fixation in photosynthetic organisms**	10	10	0.408026	0.099404	0.008099	0.251065	0.010924
**Biosynthesis of various other secondary metabolites**	4	4	0.444018	0.099404	0.008868	0.266036	0.011824
**Biosynthesis of 12-, 14- and 16-membered macrolides**	2	2	0.474415	0.099404	0.00902	0.266036	0.01189
**Folate biosynthesis**	17	17	0.402128	0.099404	0.01015	0.284199	0.013229

**Biotin metabolism**	11	11	0.393073	0.099404	0.010541	0.284614	0.013587
**Atrazine degradation**	5	5	0.47292	0.099404	0.012253	0.318565	0.015619
**Styrene degradation**	9	9	0.382586	0.099404	0.014569	0.364222	0.018369
**Glycerophospholipid metabolism**	13	13	0.390339	0.099404	0.016546	0.397096	0.020507
**Glycolysis / Gluconeogenesis**	26	26	0.370957	0.099404	0.016618	0.397096	0.020507
**Oxidative phosphorylation**	9	9	0.302385	0.099404	0.018496	0.406913	0.022585
**Monobactam biosynthesis**	4	4	0.37972	0.099404	0.023291	0.489114	0.028143
**Other glycan degradation**	3	3	0.44372	0.099404	0.02641	0.528203	0.031437
**Fatty acid biosynthesis**	7	7	0.335613	0.099404	0.026558	0.528203	0.031437
**Primary bile acid biosynthesis**	2	2	0.412556	0.099404	0.030114	0.542049	0.035285
**Phenylpropanoid biosynthesis**	3	3	0.337885	0.099404	0.046323	0.787486	0.053734
**One carbon pool by folate**	6	6	0.293231	0.099404	0.053017	0.848268	0.06089
**Metabolism of xenobiotics by cytochrome P450**	5	5	0.291296	0.099404	0.066861	1	0.076038
**Drug metabolism - cytochrome P450**	6	6	0.282668	0.099404	0.069491	1	0.078262
**Novobiocin biosynthesis**	3	3	0.258574	0.099404	0.072495	1	0.08086
**Streptomycin biosynthesis**	6	6	0.236577	0.099404	0.084969	1	0.093327
**Sphingolipid metabolism**	8	8	0.223359	0.099404	0.085282	1	0.093327
**Photosynthesis - antenna proteins**	2	2	0.196241	0.099404	0.139018	1	0.150711
**Vitamin B6 metabolism**	6	6	0.183587	0.099404	0.144439	1	0.155138
**Sesquiterpenoid and triterpenoid biosynthesis**	2	2	0.169603	0.099404	0.188799	1	0.200924
**Arachidonic acid metabolism**	2	2	0.147738	0.099404	0.21891	1	0.230851
**Lipopolysaccharide biosynthesis**	20	20	0.080997	0.099404	0.389976	1	0.407542
**Glycosaminoglycan degradation**	8	8	0.073503	0.099404	0.498703	1	0.516514
**Various types of N-glycan biosynthesis**	2	2	0.048672	0.099404	0.553885	1	0.56859
**Glycosphingolipid biosynthesis - globo and isoglobo series**	2	2	0.03788	0.099404	0.631498	1	0.642577
**Phenazine biosynthesis**	2	2	0.022335	0.099404	0.772761	1	0.77948
**Linoleic acid metabolism**	2	2	0.00921	0.099404	0.911356	1	0.911356
**Terpenoid backbone biosynthesis**	13	13	1.382153	0.099404	2.41E-07	2.77E-05	1.40E-05
**Steroid degradation**	6	6	1.500446	0.099404	2.00E-08	2.32E-06	2.32E-06
**Thiamine metabolism**	15	15	1.210224	0.099404	1.70E-06	0.000194	4.57E-05
**Benzoate degradation**	41	41	0.823567	0.099404	1.77E-06	0.0002	4.57E-05
**Polycyclic aromatic hydrocarbon degradation**	4	4	1.284818	0.099404	2.45E-06	0.000275	4.57E-05
**Biosynthesis of unsaturated fatty acids**	3	3	1.05181	0.099404	2.60E-06	0.000289	4.57E-05
**Peptidoglycan biosynthesis**	9	9	1.149397	0.099404	3.56E-06	0.000392	4.57E-05
**Glyoxylate and dicarboxylate metabolism**	41	41	0.88194	0.099404	3.90E-06	0.000425	4.57E-05
**Phenylalanine, tyrosine and tryptophan biosynthesis**	20	20	0.76523	0.099404	4.62E-06	0.000499	4.57E-05
**Methane metabolism**	41	41	0.787567	0.099404	4.90E-06	0.000524	4.57E-05
**Arginine and proline metabolism**	44	44	0.749763	0.099404	4.92E-06	0.000524	4.57E-05
**Pantothenate and CoA biosynthesis**	16	16	0.993638	0.099404	5.29E-06	0.000555	4.57E-05

**Xylene degradation**	20	20	1.199797	0.099404	5.40E-06	0.000562	4.57E-05
**C5-Branched dibasic acid metabolism**	8	8	0.883438	0.099404	5.51E-06	0.000568	4.57E-05
**Nicotinate and nicotinamide metabolism**	14	14	0.941797	0.099404	6.55E-06	0.000668	5.06E-05

PRISMA 2020 Checklist

Section and Topic	Item #	Checklist item	Location where item is reported
**TITLE**			
Title	1	Identify the report as a systematic review.	Title (reported as meta-analysis)
**ABSTRACT**			
Abstract	2	See the PRISMA 2020 for Abstracts checklist.	Abstract
**INTRODUCTION**			
Rationale	3	Describe the rationale for the review in the context of existing knowledge.	Section 1.0 Introduction and<br>Section 3.0 Discussion
Objectives	4	Provide an explicit statement of the objective(s) or question(s) the review addresses.	Section 1.0 Introduction
**METHODS**			
Eligibility criteria	5	Specify the inclusion and exclusion criteria for the review and how studies were grouped for the syntheses.	Figure 1 and<br>Section 4.2 Data Pre-processing
Information sources	6	Specify all databases, registers, websites, organisations, reference lists and other sources searched or consulted to identify studies. Specify the date when each source was last searched or consulted.	Section 4.2 Data Pre-processing
Search strategy	7	Present the full search strategies for all databases, registers and websites, including any filters and limits used.	Section 4.2 Data Pre-processing and Supplementary Table 1
Selection process	8	Specify the methods used to decide whether a study met the inclusion criteria of the review, including how many reviewers screened each record and each report retrieved, whether they worked independently, and if applicable, details of automation tools used in the process.	Figure 1 and<br>Section 4.2 Data Pre-processing
Data collection process	9	Specify the methods used to collect data from reports, including how many reviewers collected data from each report, whether they worked independently, any processes for obtaining or confirming data from study investigators, and if applicable, details of automation tools used in the process.	Section 4.2 Data Pre-processing
Data items	10a	List and define all outcomes for which data were sought. Specify whether all results that were compatible with each outcome domain in each study were sought (e.g. for all measures, time points, analyses), and if not, the methods used to decide which results to collect.	Section 4.2 Data Pre-processing and Supplementary Table 1
	10b	List and define all other variables for which data were sought (e.g. participant and intervention characteristics, funding sources). Describe any assumptions made about any missing or unclear information.	Not applicable
Study risk of bias assessment	11	Specify the methods used to assess risk of bias in the included studies, including details of the tool(s) used, how many reviewers assessed each study and whether they worked independently, and if applicable, details of automation tools used in the process.	Section 4.2 Data Pre-processing and<br>Figure 1
Effect measures	12	Specify for each outcome the effect measure(s) (e.g. risk ratio, mean difference) used in the synthesis or presentation of results.	Section 4.4 Biomarkers Detection
Synthesis methods	13a	Describe the processes used to decide which studies were eligible for each synthesis (e.g. tabulating the study intervention characteristics and comparing against the planned groups for each synthesis (item #5)).	Section 4.2 Data Pre-processing and<br>Figure 1
	13b	Describe any methods required to prepare the data for presentation or synthesis, such as handling of missing summary statistics, or data conversions.	Section 4.2 Data Pre-processing and 4.3 Diversity analysis
	13c	Describe any methods used to tabulate or visually display results of individual studies	Section 4.2 Data Pre-

Section and Topic	Item #	Checklist item	Location where item is reported
		and syntheses.	processing
	13d	Describe any methods used to synthesize results and provide a rationale for the choice(s). If meta-analysis was performed, describe the model(s), method(s) to identify the presence and extent of statistical heterogeneity, and software package(s) used.	Section 4.2 Data Pre-processing and Supplementary Table 1
	13e	Describe any methods used to explore possible causes of heterogeneity among study results (e.g. subgroup analysis, meta-regression).	Section 4.2 Data Pre-processing
	13f	Describe any sensitivity analyses conducted to assess robustness of the synthesized results.	Section 4.2 Data Pre-processing and Figure 1
Reporting bias assessment	14	Describe any methods used to assess risk of bias due to missing results in a synthesis (arising from reporting biases).	Section 4.2 Data Pre-processing and Figure 1
Certainty assessment	15	Describe any methods used to assess certainty (or confidence) in the body of evidence for an outcome.	Section 4.4 Biomarkers Detection
**RESULTS**			
Study selection	16a	Describe the results of the search and selection process, from the number of records identified in the search to the number of studies included in the review, ideally using a flow diagram.	Figure 1 and Section 2.1
	16b	Cite studies that might appear to meet the inclusion criteria, but which were excluded, and explain why they were excluded.	Supplementary Table 1
Study characteristics	17	Cite each included study and present its characteristics.	Figure 1 and Table 1 and Section 2.1
Risk of bias in studies	18	Present assessments of risk of bias for each included study.	Section 2.1
Results of individual studies	19	For all outcomes, present, for each study: (a) summary statistics for each group (where appropriate) and (b) an effect estimate and its precision (e.g. confidence/credible interval), ideally using structured tables or plots.	Supplementary Table 2 and Table 1
Results of syntheses	20a	For each synthesis, briefly summarise the characteristics and risk of bias among contributing studies.	Table 1 and Section 2.2
	20b	Present results of all statistical syntheses conducted. If meta-analysis was done, present for each the summary estimate and its precision (e.g. confidence/credible interval) and measures of statistical heterogeneity. If comparing groups, describe the direction of the effect.	Supplementary Table 2 and Section 2.5
	20c	Present results of all investigations of possible causes of heterogeneity among study results.	Figure 1 and Section 2.1 and Supplementary Table 1
	20d	Present results of all sensitivity analyses conducted to assess the robustness of the synthesized results.	Figure 1 and Section 2.1 and 2.2
Reporting biases	21	Present assessments of risk of bias due to missing results (arising from reporting biases) for each synthesis assessed.	Figure 1 and Section 2.1
Certainty of evidence	22	Present assessments of certainty (or confidence) in the body of evidence for each outcome assessed.	Figure 1 and Section 2.1 and Supplementary Table 1
**DISCUSSION**			
and Discussion	23a	Provide a general interpretation of the results in the context of other evidence.	Section 5.0 Discussion and Section 1.0 Introduction
	23b	Discuss any limitations of the evidence included in the review.	Section 5.0 Discussion and

Section and Topic	Item #	Checklist item	Location where item is reported
			Supplementary Table 1
	23c	Discuss any limitations of the review processes used.	Section 5.0 Discussion
	23d	Discuss implications of the results for practice, policy, and future research.	Section 5.0 Discussion and Section 1.0 Introduction
**OTHER INFORMATION**			
Registration and protocol	24a	Provide registration information for the review, including register name and registration number, or state that the review was not registered.	Not applicable
	24b	Indicate where the review protocol can be accessed, or state that a protocol was not prepared.	Section 5 Data availability
	24c	Describe and explain any amendments to information provided at registration or in the protocol.	Not applicable
Support	25	Describe sources of financial or non-financial support for the review, and the role of the funders or sponsors in the review.	Section 7 Acknowledgement
Competing interests	26	Declare any competing interests of review authors.	Section 9 Competing interests
Availability of data, code and other materials	27	Report which of the following are publicly available and where they can be found: template data collection forms; data extracted from included studies; data used for all analyses; analytic code; any other materials used in the review.	Table 1 and Section 5 Data availability and Section 6 Code availability

From: Page MJ, McKenzie JE, Bossuyt PM, Boutron I, Hoffmann TC, Mulrow CD, et al. The PRISMA 2020 statement: an updated guideline for reporting systematic reviews. BMJ 2021;372:n71. doi: 10.1136/bmj.n71. This work is licensed under CC BY 4.0. To view a copy of this license, visit https://creativecommons.org/licenses/by/4.0/

PRISMA 2020 for Abstracts Checklist

Section and Topic	Item #	Checklist item	Reported (Yes/No)
**TITLE**			
Title	1	Identify the report as a systematic review.	Yes (reported as meta-analysis)
**BACKGROUND**			
Objectives	2	Provide an explicit statement of the main objective(s) or question(s) the review addresses.	Yes
**METHODS**			
Eligibility criteria	3	Specify the inclusion and exclusion criteria for the review.	Yes
Information sources	4	Specify the information sources (e.g. databases, registers) used to identify studies and the date when each was last searched.	Yes
Risk of bias	5	Specify the methods used to assess risk of bias in the included studies.	Yes
Synthesis of results	6	Specify the methods used to present and synthesise results.	Yes
**RESULTS**			
Included studies	7	Give the total number of included studies and participants and summarise relevant characteristics of studies.	Yes
Synthesis of results	8	Present results for main outcomes, preferably indicating the number of included studies and participants for each. If meta-analysis was done, report the summary estimate and confidence/credible interval. If comparing groups, indicate the direction of the effect (i.e. which group is favoured).	Yes
**DISCUSSION**			
Limitations of evidence	9	Provide a brief summary of the limitations of the evidence included in the review (e.g. study risk of bias, inconsistency and imprecision).	No
Interpretation	10	Provide a general interpretation of the results and important implications.	Yes
**OTHER**			
Funding	11	Specify the primary source of funding for the review.	Yes
Registration	12	Provide the register name and registration number.	No

From: Page MJ, McKenzie JE, Bossuyt PM, Boutron I, Hoffmann TC, Mulrow CD, et al. The PRISMA 2020 statement: an updated guideline for reporting systematic reviews. BMJ 2021;372:n71. doi: 10.1136/bmj.n71. This work is licensed under CC BY 4.0. To view a copy of this license, visit https://creativecommons.org/licenses/by/4.0/
